# Enzalutamide inhibits *PEX10* function and sensitizes prostate cancer cells to ROS activators

**DOI:** 10.1038/s41419-024-06937-7

**Published:** 2024-08-03

**Authors:** Yuankang Feng, Yu Zhang, Hao Li, Tao Wang, Fubo Lu, Ruoyang Liu, Guoqing Xie, Liang Song, Budeng Huang, Xiang Li, Yinghui Ding, Jinjian Yang, Zhankui Jia, Zhenlin Huang

**Affiliations:** Department of Urology, The First Affiliated Hospital of Zhengzhou University, Zhengzhou, 450052 China

**Keywords:** Prostate cancer, Transcriptional regulatory elements

## Abstract

Sharply increased reactive oxygen species (ROS) are thought to induce oxidative stress, damage cell structure and cause cell death; however, its role in prostate cancer remains unclear. Enzalutamide is a widely used anti-prostate cancer drug that antagonizes androgen binding with its receptor. Further exploration of the mechanism and potential application strategies of enzalutamide is crucial for the treatment of prostate cancer. Here, we confirmed *PEX10* can be induced by ROS activators while reduce ROS level in prostate cancer cells, which weakened the anti-tumor effect of ROS activators. The androgen receptor (AR) can promote the expression of *PEX10* by acting as an enhancer in cooperation with FOXA1. The anti-tumor drug enzalutamide inhibits *PEX10* by inhibiting the function of AR, and synergize with ROS activators ML210 or RSL3 to produce a stronger anti-tumor effect, thereby sensitizing cells to ROS activators. This study reveals a previously unrecognized function of enzalutamide and AR by regulating *PEX10* and suggests a new strategy of enzalutamide application in prostate cancer treatment.

## Introduction

Prostate cancer is the most lethal cancer in men, with a high incidence and mortality rate [1–3]. Due to the lack of obvious symptoms in the early stages of prostate cancer, it is often detected after metastasis, particularly to the bones, which is a significant cause of patient mortality [4, 5]. Current treatments include androgen inhibition-based comprehensive therapy, however, more than 70% of prostate cancer patients develop castration-resistant prostate cancer (CRPC) [6, 7]. Novel therapeutics, including enzalutamide, offer new treatment opportunities for patients with CRPC. Enzalutamide, as a novel anti-androgen medication, is primarily used to treat advanced castration-resistant prostate cancer that has spread or recurred. However, the decreased sensitivity to enzalutamide limits its application and complicates advanced CRPC treatment [8, 9]. In patients with CRPC, resistance to enzalutamide involves several aspects, including AR gene duplication [10, 11], AR splice variants (AR-Vs) [12], AR gene mutations [13] and upregulation of aberrant glucocorticoid receptor (GR) [14]. Increasing numbers of clinical practices try to expanding the clinical application indications of enzalutamide through combination, and expected it could achieve a better therapeutic effects. For example, the FDA approved enzalutamide combine Talzenna for the treatment of metastatic castration-resistant prostate mCRPC with homologous recombination repair (HRR) gene mutations [15]. Therefore, the exploration of the combination of enzalutamide will be a promising study.

Reactive Oxygen Species (ROS), often denoted as free radicals, constitute a class of highly reactive molecules or ions characterized by unpaired electrons [16]. They are generated within various cell organelles, including peroxisomes and mitochondria, and play a dual role in cancer development. Initially, they play crucial roles in regular physiological processes, including bolstering the immune system and facilitating signal transduction, thereby maintaining the equilibrium of life [17]. Nevertheless, when the generation of ROS surges or the mechanisms responsible for their elimination fall into imbalance, they may transform into deleterious entities. In certain scenarios, malignant cells can generate an excessive quantity of ROS, thereby stimulating cellular proliferation and survival [18]. Meanwhile, in numerous studies, it has been demonstrated that ROS can be leveraged to combat tumors by inducing cell cycle arrest or apoptosis.

PEX10, a member of the PEX family, is primarily involved in the lipid oxidation pathway and participates in forming peroxisomes [19]. Many genes involved in peroxisome formation [20], including *PEX10*, play important roles in maintaining ferroptosis sensitivity in various tumor cells [21]. A close association exists between reactive oxygen species and peroxisomes. Peroxisomes are cellular organelles housing a variety of enzymes, including one known as catalase. Catalase plays an essential role within cells in the elimination of reactive oxygen species [22]. Interestingly, data from the Cancer Genome Atlas (TCGA) shows that *PEX10* expression is markedly elevated in prostate cancer and closely associated with lymph node metastasis of prostate cancer. This finding suggests that *PEX10* may plays a unique and important role in prostate cancer.

In this study, we demonstrate that AR promotes *PEX10* expression and downregulate ROS level. In contrast, enzalutamide inhibits *PEX10* expression and eventually increase ROS anti-tumor function in prostate cancer. Our study reveals the mechanism by which the combination of ROS activator and enzalutamide can help increase RSL3 or ML210 drug sensitivity and achieve better inhibition effects in prostate cancer, thereby providing a new strategy for prostate cancer therapy.

## Materials and methods

### Data mining

The Cancer Genome Atlas Program (TCGA; https://portal.gdc.cancer.gov/) [23] dataset was used to analyze the expression of PEX10 and its correlation with AR and FOXA1, with data visualization conducted using the Gene Expression Profiling Interactive Analysis (GEPIA; http://gepia.cancer-pku.cn/) [24] database and R-4.4.1 (https://www.r-project.org/). Gene Set Enrichment Analysis (GSEA; https://www.gsea-msigdb.org/gsea/index.jsp) [25, 26] and Kyoto Encyclopedia of Genes and Genomes (KEGG; https://www.genome.jp/kegg/) [27] were employed to analyze the signaling pathways affected by ML210. Finally, the PPI protein network was constructed using the IntAct (https://www.ebi.ac.uk/intact/home) [28], GENEMANIA (https://genemania.org/) [29] and STRING (https://string-db.org/) [30] databases.

### Cell lines, cell culture, and transfection

The LNCaP, C4-2, DU145, PC-3, 22Rv1, and 293 T cell lines were purchased from the Cell Bank of the Chinese Academy of Sciences (Shanghai, China) (Supplementary Table S1). LNCaP, C4-2, and 22Rv1 cells were cultured in RPMI-1640 medium supplemented with 10% FBS. 293 T cells were cultured in DMEM medium supplemented with 10% FBS. DU145 cells were cultured in MEM medium supplemented with 10% FBS. PC-3 cells were cultured in Ham’s F-12 medium supplemented with 10% FBS. The cells were cultured for use in the logarithmic growth phase. Lipofectamine 2000 (Thermo Fisher Scientific, Waltham, MA, USA) was used for cell transfection, following the manufacturer’s protocol (shRNA sequence information in Supplementary Table S2). All transfection operations were performed in six-well plates, with 5 μl of Lipofectamine 2000 used per well for plasmid transfection.

Cultivate C4-2 cells in medium containing enzalutamide, sequentially increasing the concentration of enzalutamide in complete culture medium (1-20 μM). Maintain the culture for 2 months after reaching a concentration of 5 μM to obtain an enzalutamide-resistant C4-2 cell line.

### ROS assay

The intracellular ROS content was complemented and validated using an H2DCFDA (DCFH-DA, DCFH) ROS fluorescence probe (MKBio, Shanghai, China). We plated 2.5 × 10^5^ cells on a 6-well plate, and the culture medium was extracted before adding a 10 mM working solution with PBS. The plate was incubated at 25 °C for 40 min, following which the working staining solution was removed, and the plate was washed once with a cell culture medium. The cell culture medium was re-added to the cells, and the ROS staining level was evaluated with a microscope (Leica DMIRB, Weztlar, Germany).

### RNA sequencing (RNA-seq)

Total RNA was extracted using TRIzol reagent (Invitrogen, Carlsbad, CA, USA) following the manufacturer’s instructions. The total RNA concentration and purity were determined using a Bioanalyzer 2100 and RNA 6000 Nano LabChip Kit (Agilent, Santa Clara, CA, USA), Renewable identification numbers (RIN) > 7.0. Poly (A) mRNA was isolated using poly T oligo-attached magnetic beads (Invitrogen, Waltham, MA, USA), and approximately 10 µg of total RNA representing specific fat types was extracted. After purification, the poly (A) – or poly (A) + RNA fragments were decomposed into smaller fragments using divalent cations at high temperatures. The RNA fragments were then reverse-transcribed according to the instructions of the Illumina sample preparation kit (Illumina, San Diego, CA, USA) to generate a final cDNA library with an average insertion size of 300 bp (±50 bp) for paired-end libraries. Subsequently, we performed paired-end sequencing on Illumina HiSeq 4000 (lc-bio, Hangzhou, China) following the vendor’s recommended protocol.

### Western blotting analysis

RIPA buffer (R0010; Solarbio, Beijing, China) was used to lyse the cells, and protein concentrations were determined using a bicinchoninic acid protein assay kit (Beijing Leagene Biotech Co, Ltd., Beijing, China). Protein samples (25–40 µg/lane) were separated by sodium dodecyl-sulfate polyacrylamide gel electrophoresis on 10% gels and transferred to polyvinylidene fluoride (PVDF) membranes. The membranes were blocked using a protein-free rapid block buffer (Epizyme Pharmaceutical Biotechnology Co, Ltd, Shanghai, China) for 15 min at 25 °C and then incubated with primary antibodies overnight at 4 °C, followed by secondary antibodies at 25 °C for 1 h. The membranes were then scanned using an imaging system (ODYSSEY ® CLx, Gene Company Limited, Lincoln, NE, USA), and densitometry was quantified using Image Studio Lite (LI-COR Biosciences, Lincoln, NE, USA) (Supplementary Table S1).

### Immunohistochemistry

The paraffin sections from human prostate cancer tissues or CRPC organoid were deparaffinized in xylene solution for 10 min. Hydrogen peroxide was added to block endogenous peroxidase, and the tissues were incubated at 25 °C for 10 min in the dark, rinsed with distilled water for 5 min, and then subjected to antigen retrieval treatment. Next, after rinsing with PBS for 5 min, the tissues were combined with serum homologous to the secondary antibody and then placed in a 37 °C environment for enclosed treatment for 15 min. The tissues were incubated with primary antibody at 4 °C overnight and washed with PBS for 5 min. Then, chelates containing secondary antibodies were added, labeled with horseradish peroxidase, and incubated at 37°C for 40 min, followed by washing with PBS for 5 min. Finally, the 3, 3’-Diaminobenzidine (DAB) staining solution was added for rendering color rendering. Staining results were observed under a microscope (Leica DMIRB, Weztlar, Germany). Rabbit monoclonal anti-AR (1:500, ab133273, Abcam), Rabbit polyclonal anti-PEX10 (1:100, PA5-116706, Thermofisher Scientific).

### Senescence associated β-galactosidase assay

For in vitro experiment, SA-β-gal staining was performed using the Senescence b-Galactosidase Staining Kit (Beyotime, Shanghai, China) according to the manufacturer’s instructions.

### Chromatin immunoprecipitation (ChIP)-qPCR

A high-sensitivity ChIP kit (ab185913; Abcam) was used to perform ChIP-qPCR according to the manufacturer’s instructions. The samples were homogenized and crosslinked using 1% formaldehyde and then reacted with 1.25 M glycine. After centrifugation, the pellet was homogenized with a working lysis buffer and added to the ChIP buffer provided in the kit. Chromatin was sheared into fragments of approximately 300 bp by sonication, and fragmentation was verified by agarose gel electrophoresis. After centrifugation, the working lysis buffer and the ChIP buffer supplied in the kit were added; chromatin was then cut into approximately 300 bp segments by ultrasound and verified by agarose gel electrophoresis. Immunoprecipitation was performed using ChIP-grade antibodies and non-immune IgG as negative controls. Small equal portions of the immune complex and lysate (as input controls) were treated with the DNA release buffer provided in the kit and protease K to reverse crosslinking and purify the DNA. The purified DNA was used as an input sample in qPCR, as detected by the PROMO software online tool. The SsoAdvanced Universal SYBR Green Supermix (Bio-Rad Laboratories, Hercules, CA, USA) and CFX Connect Real-Time PCR detection system equipped with the CFX manager software (Bio-Rad Laboratories) were used. Fold enrichment was calculated using the Ct method using the ratio of amplification efficiency of the ChIP sample to that of non-immune IgG (fold enrichment = 2(IgG Ct − sample Ct)). Rabbit monoclonal anti-AR (Use 5 µg for 25 µg of chromatin, ab108341, Abcam) and, Rabbit monoclonal anti-FOXA1 (Use 5 µg for 25 µg of chromatin, ab170933, Abcam) were used.

### Organoid construction and culture

The CRPC Prostate tissue obtained from the First Affiliated hospital of Zhengzhou University were selected and cut into pieces for digestion and dissociation at 37 °C for 1 h. Cell mass was then cultured in a 3D environment using DMEM/F-12 (Gibco, Grand Island, NE, USA), Matrigel (356234; Corning, Corning, CA, USA), serum substitutes (B27) (Thermo Fisher Scientific, Waltham, MA, USA), small molecule inhibitors (A83-01 and Y-27632) (MedChemExpress, New Jersey, United States), and growth factors (Activin A, RSPO1, EGF, FGF-10, Noggin, and FGFb) (Novoprotein, Shanghai, China) for growth into organoids. When the subculture was carried out, the medium was first discarded, the Matrigel was blown with pre-cooled Matrigel recovery solution, the recovery solution was discarded by centrifugation, and the Organoid Dissociation Solution (Mogengel, Xiamen, China) digestive solution was added for 10 min. Digestion was terminated when most organoids were dissociated into 3–10 cell clusters. The tissue and epithelial original specificity of the organoid were identified by IHC of AR and CK5/6. The organoids growth was measured with microscope (Leica DMIRB, Weztlar, Germany). The study design was approved by the Ethics Committee of the First Affiliated Hospital of Zhengzhou University (2022-KY-0239-002).

### In vivo tumor xenograft model

Age-matched 6-week-old male severe combined immunodeficiency (SCID) mice were purchased from the Sipeifu Company (Beijing, China). To meet statistical requirements, 40 mice were randomly divided into eight groups (5 mice per group) to minimize experimental error. Then, 1×10^6^ C4-2 cells with or without *PEX10* stable overexpression were suspended in the Matrigel (Becton, Dickinson and Company, Franklin Lakes, NJ, USA) in a 1:1 ratio, extracted with a 1-mL syringe, and injected into the subcutaneous at a 45° angle. The mice with established tumors with ~50–80 mm^3^ average volumes (measured by calipers and calculated as (length × width × height) / 2) were randomized into treatment groups including vehicle (DMSO), ML210 (5 mg/kg in 20 μl DMSO plus 130 μl corn oil, ip, daily). For combination therapy experiments, mice were randomized into different treatment groups including vehicle, ML210 (5 mg/kg), enzalutamide (10 mg/kg in 5% DMSO, 30% PEG 300, 65% H_2_O, oral gavage, daily). After 33 days, the mice were sacrificed, and the tumor masses were removed and photographed. The mice experiments were approved by the Ethics Committee of the First Affiliated Hospital of Zhengzhou University (ZZU-LAC20210924).

### Statistical analysis

Data are expressed as means ± SD from at least three independent experiments. All data were analyzed using GraphPad Prism 7.0 (La Jolla, CA, USA). Either the Student’s t-test or ANOVA was used to analyze the groups unless otherwise indicated. Statistical significance was set at *P* < 0.05.

## Results

### *PEX10* is involved in ROS regulation in prostate cancer

**Fig. 1:**
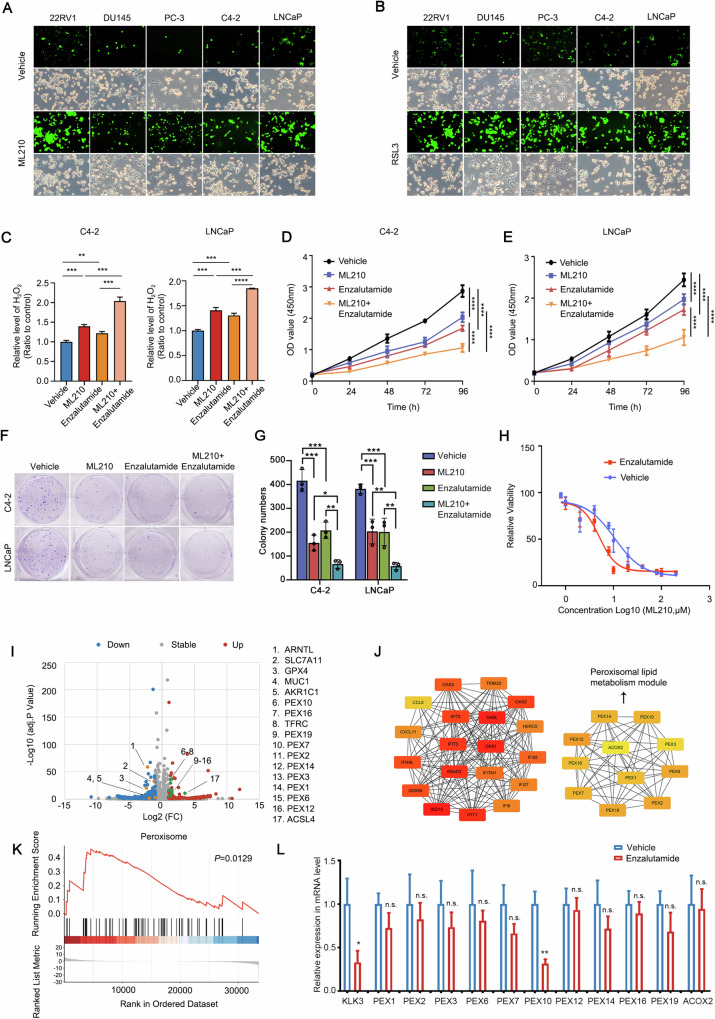
PEX10 is involved in ROS in prostate cancer. **A**, **B** Prostate cancer cell lines (22Rv1, DU145, PC-3, C4-2, and LNCaP) treated with vehicle or ML210 (2 μM) (**A**) or RSL3 (1 μM) (**B**) and evaluation of the cell count and cell ROS level according to manufacturer’s instructions. **C** H_2_O_2_ level of prostate cancer cells (C4-2 and LNCaP) after treatment with ML210 (2 μM) or enzalutamide (5 μM) or combination. Unpaired t-test. (*P* < 0.0001 as “****”). **D**, **E** The CCK-8 OD value of two cell lines (C4-2-R and C4-2-N) after treating with ML210 (2 μM) and with or without enzalutamide (5 μM) combination. ANOVA. (*P* < 0.01 as “**”; *P* < 0.001 as “***”; *P* < 0.0001 as “****”). **F**, **G** Colony formation assay were performed on two cell lines (C4-2-R and C4-2-N) after treated with ML210 (2 μM) alone or in combination with enzalutamide (5 μM). Unpaired t-test. (*P* < 0.05 as “*”; *P* < 0.01 as “**”; *P* < 0.001 as “***”). **H** The IC50 of ML210 in prostate cell lines (C4-2) with or without enzalutamide (5 μM) treatment. **I**–**K** The images represented the results of C4-2 sequencing processed by ML210 (2 μM) or vehicle. Three independent samples were taken from each group and treated with ML210 (2 μM) or vehicle for 48 h before sampling. The volcano showed up- and down-regulated genes after ML210 treatment (**I**). The result of PPI showed that the peroxisome-related genes had changed significantly (**J**). Peroxisome pathway was the most significantly changed pathway after ML210 treatment in GSEA analysis (**K**). **L** The expression of Peroxisome-related genes and other key genes (KLK3) in prostate cancer cells (C4-2) after treatment with enzalutamide (5 μM). Unpaired t-test. (*P* < 0.05 as “*”; *P* < 0.01 as “**”; n.s. means nonspecific).

In many cancer types, including ovarian, rectal, and breast cancers, reactive oxygen species (ROS) remarkably inhibits cancer cell proliferation [20, 31–34]; however, its detailed mechanism in prostate cancer remains unclear. To further explore the role and detailed functional mechanism of ROS regulation in prostate cancer, we treated various prostate cancer cell lines with ROS inducers, ML210 and RSL3, and detected ROS and cell proliferation levels. Both ML210 and RSL3 significantly increased ROS levels (Figs. 1A, B and S1A) and significantly inhibited the proliferation of several prostate cancer cell lines (Fig. S1B–C). These results suggest that ROS inducers can significantly increase ROS production and inhibit prostate cancer cell proliferation. Due to the inclusion of Enzalutamide in our experimental drug research, we have selected LNCaP and C4-2 as the focal points of our study.

Furthermore, our observations revealed that the combination of enzalutamide with a ROS inducer yielded superior H_2_O_2_ activation (Fig. 1C) and more effective inhibition of proliferation (Fig. 1D–G) in both C4-2 and LNCaP cells compared to the administration of enzalutamide or ML210 treatment alone. Meanwhile, PMP70 levels, which reflect peroxisome levels, decreased significantly (Fig. S1D and E). In the other hand, enzalutamide can significantly reduce the IC50 of ML210 in prostate cancer cells, which indicated that enzalutamide enhanced the tumor growth inhibition effect of ML210 in prostate cancer (Fig. 1H).

To further understand the mechanism of ROS in prostate cancer, we treated C4-2 cells with ML210 and performed RNA-seq to analyze the ROS pathway genes of in prostate cancer. RNA-seq data detected 178 upregulated and 415 downregulated genes (adj *P* < 0.05, |Log2FC | > 1) (Fig. 1I). *GPX4* was significantly downregulated, whereas *ACSL4* were significantly upregulated, confirming the effectiveness of the drug treatment. Peroxisome family genes, which participate in peroxisome formation, were significantly upregulated after ML210 treatment in C4-2 cells, which is consistent with a previous study on renal and ovarian cancers [21]. We performed PPI analysis on differential expression genes and extracted hub genes and found the peroxisome pathways significantly changed (Fig. 1J). Gene Set Enrichment Analysis (GSEA) and Kyoto Encyclopedia of Genes and Genomes (KEGG) pathway enrichment also showed that the peroxisome pathway was the most significantly altered pathway, thereby suggesting that peroxisome genes may play a crucial role in ROS regulation in prostate cancer (Figs. 1K and S1F).

To assess the impact of enzalutamide on the expression of peroxisome-related genes, we conducted an analysis of differentially expressed genes following enzalutamide administration. Remarkably, among all peroxisome genes, only *PEX10* exhibited significant downregulation after enzalutamide treatment (Fig. 1L). These findings underscore the pivotal role of *PEX10* in mediating enzalutamide’s regulation of peroxisome function and the ROS processes in prostate cancer.

### *PEX10* promote cell proliferation by eliminating ROS in prostate cancer

**Fig. 2:**
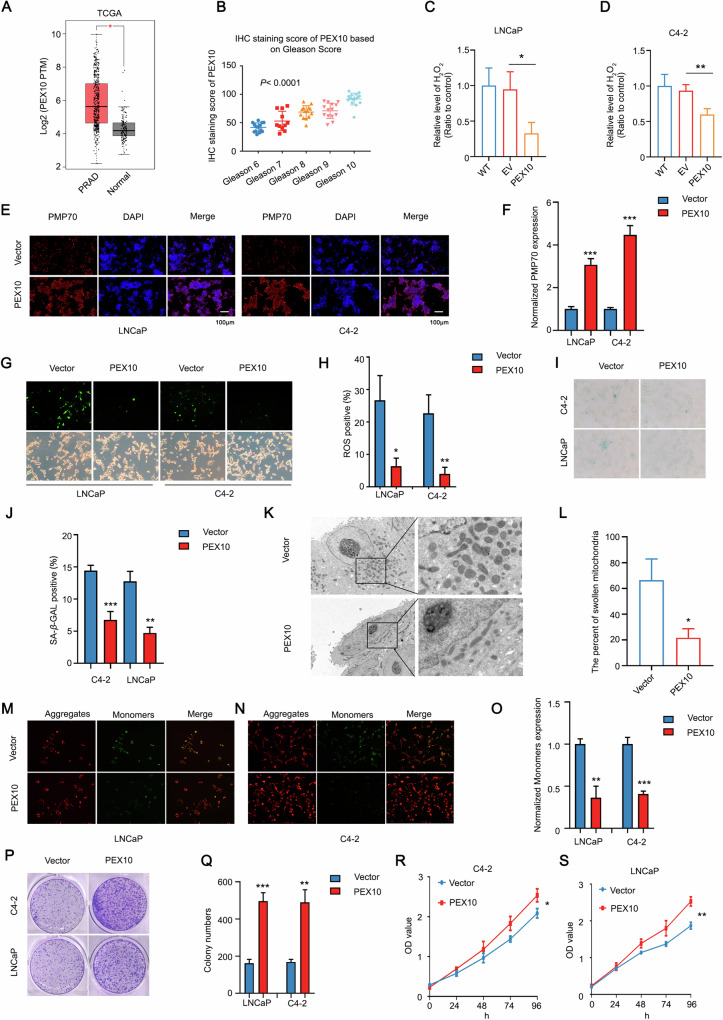
PEX10 suppresses cell death by decreasing ROS and H_2_O_2_. **A** Expression of *PEX10* in prostate cancer and normal tissues from the TCGA database. Unpaired t-test. (*P* < 0.05 as “*”). **B** The image showed the IHC staining score of PEX10 based on Gleason Score. One-way ANOVA. IHC staining score = ∑ (pi×i) = (percentage of weak intensity area ×1) + (percentage of moderate intensity area ×2) + (percentage of strong intensity area ×3), pi represents the percentage of the area of positive signal pixels; i represents the positive grade. **C**, **D** H_2_O_2_ level in prostate cancer cells (C4-2 and LNCaP) after overexpression of *PEX10*. Unpaired t-test. (*P* < 0.05 as “*”; *P* < 0.01 as “**”). **E**, **F** PMP70 expression level in prostate cancer cells (C4-2 and LNCaP) after overexpression of *PEX10*. Unpaired t-test. (*P* < 0.001 as “***”). **G**, **H** ROS level in prostate cancer cells (C4-2 and LNCaP) after overexpression of *PEX10*. Unpaired t-test. (*P* < 0.001 as “***”). **I**, **J** SA-β-GAL level in prostate cancer cells (C4-2 and LNCaP) after overexpression of *PEX10*. Unpaired t-test. (*P* < 0.05 as “*”; *P* < 0.01 as “**”). **K**, **L** The percentage of mitochondria with atrophy and increased membrane density and its statistic in C4-2 cells after PEX10 ectopic expression. Unpaired t-test. (*P* < 0.05 as “*”). **M**–**O** The immunofluorescence staining of JC-1 and statistic in prostate cancer cells (C4-2 and LNCaP) after overexpression of *PEX10*. Unpaired t-test. (*P* < 0.01 as “**”, *P* < 0.001 as “***”). **P**, **Q** Colony numbers and statistic in prostate cancer cells (C4-2 and LNCaP) after overexpression of *PEX10*. Unpaired t-test. **(***P* < 0.01 as “**”; *P* < 0.001 as “***”). **R**, **S** CCK8 OD value and statistic in prostate cancer cells (C4-2 and LNCaP) after overexpression of *PEX10*. ANOVA. (*P* < 0.05 as “*”; *P* < 0.01 as “**”).

In order to further explore the role of PEX10 in prostate cancer and enzalutamide resistant prostate cancer, we first analyzed the expression of PEX10 in the TCGA database, and we found that the expression level of PEX10 was significantly increased in prostate cancer tissues (Fig. 2A), and its expression level was significantly positively correlated with Gleason score (Fig. 2B), subsequently, we obtained the same results in patient specimens (Fig. S2A, B). Peroxisomes are postulated to mitigate cellular damage by eliminating surplus ROS, thereby shielding cells from potential harm. The extent of *PEX10*’s involvement in peroxisomal function within the context of prostate cancer and its influence on ROS levels remains ambiguous. In the H_2_O_2_ experiment, ectopic overexpression of *PEX10* demonstrated a substantial inhibition of H_2_O_2_ expression in both C4-2 and LNCaP cells. (Fig. 2C, D).

Consistently, fluorescence experiments showed that overexpression *PEX10* could significantly increase the expression level of peroxisome marker PMP70 (Fig. 2E–H).

It has been verified that ROS play a role in the cellular aging process and concurrently impede the viability of cancer cells. SA-β-gal staining revealed that overexpression of *PEX10* significantly downregulated the SA-β-gal level in both C4-2 and LNCaP cells. (Fig. 2I, J), which suggest that *PEX10* may also involve in the cellular senescence induced by ROS in prostate cancer cells. Correspondingly, by observing mitochondrial membrane potential under electron microscope and JC-1 level, we found that mitochondrial membrane potential decreased significantly after *PEX10* ectopically transfection (Fig. 2K–O), which was consistent with the its role in eliminating ROS. Consistent with the previous results, both colony formation and CCK8 assay affirm that the overexpression of *PEX10* in various prostate cancer cells markedly enhances cell proliferation (Fig. 2P–S), suggesting that PEX10 plays a role in promoting cell proliferation in prostate cancer.

To further demonstrate that the ROS generated by knocking down *PEX10* can promote senescence in prostate cancer cells, we conducted additional experiments. Firstly, following *PEX10* knockdown, we supplemented with the ROS inhibitor NAC and observed a significant reduction in ROS levels produced by *PEX10* knockdown in prostate cancer cells (Fig. S2C). Subsequent β-galactosidase staining showed that senescence in prostate cancer cells was also inhibited when ROS was suppressed (Fig. S2D). This suggests that the increased level of senescence caused by knocking down *PEX10* in prostate cancer cells is mediated through elevated ROS levels. Additionally, colony formation and CCK-8 assays further confirmed that ROS and cellular senescence in prostate cancer lead to cell death (Fig. S2E, F). We also used a telomerase inhibitor BIBR 1532 alongside *PEX10* overexpression. The results showed that the telomerase inhibitor BIBR 1532 could reverse the reduced senescence caused by *PEX10* overexpression (Fig. S2G). Additionally, colony formation and CCK-8 assays indicated that when senescence was reactivated in prostate cancer cells, cell death significantly increased (Fig. S2H, I).

These experiments indicate that reactive oxygen species (ROS) lead to oxidative stress and cellular senescence, which consequently cause prostate cancer cell death.

### Knocking down *PEX10* enhances the anticancer efficiency of ML210

**Fig. 3:**
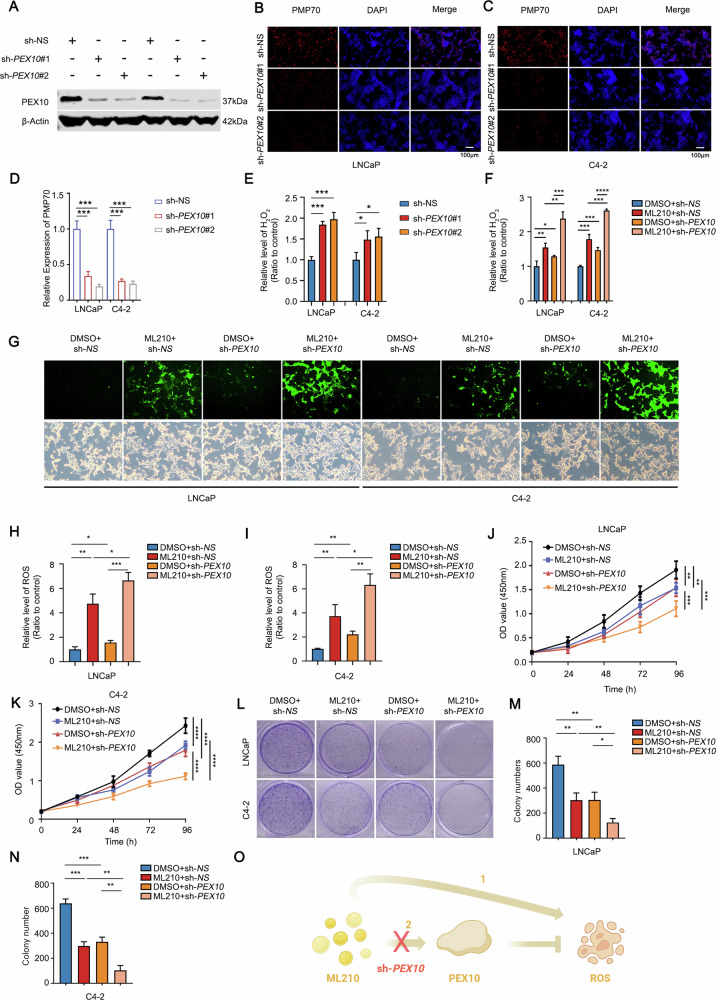
Knocking down PEX10 enhances the anticancer efficacy of ML210. **A** The expression of PEX10 after knockdown of *PEX10*. **B**–**D** PMP70 expression level in prostate cancer cells (C4-2 and LNCaP) after *PEX10* knockdown. Unpaired t-test. (*P* < 0.001 as “***”). **E**, **F** H_2_O_2_ level in prostate cancer cells (C4-2 and LNCaP) after *PEX10* knockdown. Unpaired t-test. (*P* < 0.05 as “*”; *P* < 0.01 as “**”). **G**–**I** ROS level in prostate cancer cells (C4-2 and LNCaP) after treating with ML210 (2 μM) and (or) *PEX10* knockdown. Unpaired t-test. (*P* < 0.05 as “*”; *P* < 0.01 as “**”, *P* < 0.001 as “***”). **J**, **K** CCK8 OD value and statistic in prostate cancer cells (C4-2 and LNCaP) after treating with ML210 (2 μM) and (or) *PEX10* knockdown. ANOVA. (*P* < 0.01 as “**”; *P* < 0.001 as “***” ; *P* < 0.0001 as “****”). **L**–**N** Colony numbers and statistic in prostate cancer cells (C4-2 and LNCaP) after treating with ML210 (2 μM) and (or) *PEX10* knockdown. Unpaired t-test. (*P* < 0.05 as “*”; *P* < 0.01 as “**”; *P* < 0.001 as “***”). **O** Specific mechanistic diagram of the ML210/*PEX10*/ROS axis. Created with BioRender.com (www.biorender.com).

To further investigate the role of *PEX10* in ferroptosis and prostate cancer proliferation, we downregulated *PEX10* in both LNCaP and C4-2 cells. Reduced *PEX10* expression was observed to downregulate the PMP70 level (Fig. 3A–D) and elevate H_2_O_2_ levels (Fig. 3E), aligning with the outcomes observed in the overexpression experiments. In the previous results, we confirmed that enzalutamide combined with ML210 can achieve a better inhibitory effect on tumor cell proliferation, and PEX10 may play an important role in this process. As a constituent of peroxisome genes, *PEX10* participates in the generation of peroxisomes and the mitigation of ROS. Consequently, we hypothesized that the activation of *PEX10* by ML210 diminishes intracellular ROS levels, while enzalutamide, by inhibiting *PEX10* expression, hinders this process. The synergistic administration of these drugs is postulated to achieve a more pronounced inhibitory effect on cell proliferation. To validate this mechanism, we treated cells with ML210, with or without *PEX10* knockdown, and assessed intracellular H_2_O_2_ and ROS levels. The results indicated a substantial increase in both H_2_O_2_ (Fig. 3F) and ROS levels (Fig. 3G–I) following ML210 treatment. Importantly, this effect was further enhanced upon *PEX10* knockdown (Fig. 3F–I). Furthermore, corroborating evidence from both CCK8 (Fig. 3J, K) and colony formation assays (Fig. 3L–N) affirmed that the combination of ML210 and *PEX10* knockdown achieved superior inhibition of tumor cell growth compared to ML210 or *PEX10* knockdown alone. The incorporation of ML210 and the overexpression of *PEX10* in prostate cancer provide additional substantiation for this observation (Fig. S3A–C). These findings suggest that the elevation of PEX10 induced by ML210 partially impedes ROS and diminishes its anti-tumor proliferation efficacy to a certain extent. Conversely, the suppression of PEX10 could amplify the anti-tumor impact of ML210 (Fig. 3O).

### AR play a critical role in the regulation of *PEX10* expression and function

**Fig. 4:**
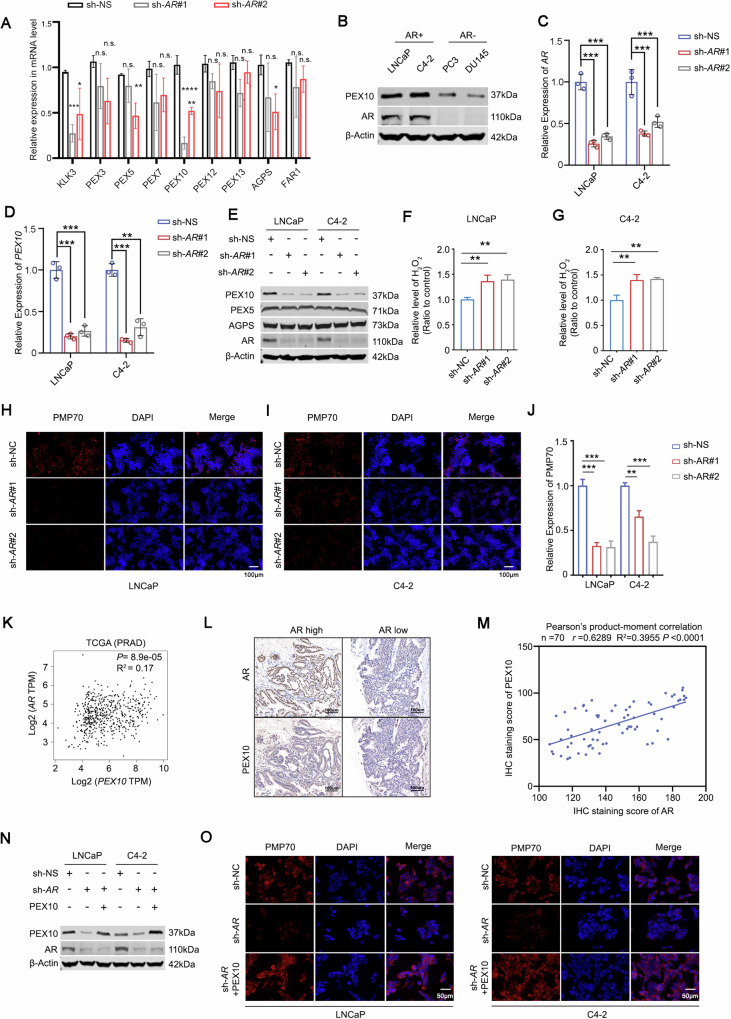
AR plays a critical role in regulation of PEX10 expression and function. **A** The expression of Peroxisome-related genes and other key genes (*KLK3*, *AGPS* and *FAR1*) in prostate cancer cells (C4-2) after *AR* knockdown. Unpaired t-test. (*P* < 0.05 as “*”; *P* < 0.01 as “**”; *P* < 0.001 as “***”; *P* < 0.0001 as “****”). **B** The protein level of PEX10 in AR-positive prostate cancer cells (C4-2 and LNCaP) and AR-negative prostate cancer cells (PC-3 and DU145). **C**, **D** *AR* and *PEX10* expression at mRNA level in prostate cancer cells (C4-2 and LNCaP) after *AR* knockdown, Unpaired t-test. (*P* < 0.01 as “**”; *P* < 0.001 as “***”). **E** AR, PEX5, AGPS and PEX10 expression at the protein level (C4-2 and LNCaP) after *AR* knockdown. **F**, **G** H_2_O_2_ level in prostate cancer cells (C4-2 and LNCaP) after *AR* knockdown. Unpaired t-test. (*P* < 0.01 as “**”). **H**–**J** PMP70 expression level in prostate cancer cells (C4-2 and LNCaP) after *AR* knockdown. Unpaired t-test. (*P* < 0.01 as “**”, *P* < 0.001 as “***”). **K**–**M** The correlation of *AR* and *PEX10* expression from the TCGA cohort (**K**). IHC shows the correlation of *AR* and *PEX10* expression in prostate cancer patient tissues (*n* = 70, r = 0.6289, R2 = 0.3955, *P* < 0.0001) (**L**, **M**). **N** The expression of PEX10 and AR protein after *AR* knockdown or combined with ectopic expression of *PEX10* in prostate cancer cells (C4-2 and LNCaP). **O** The expression of PMP70 protein after *AR* knockdown or combined with ectopic expression of *PEX10* in prostate cancer cells (C4-2 and LNCaP).

Enzalutamide significantly affected *PEX10* expression, and we explored whether it was mediated by AR. We knocked down *AR* in C4-2 cells and examined the expression of peroxisome pathway genes. Consistent with previous results, the *PEX10* was most significantly downregulated after *AR* knockdown (Fig. 4A). We also found that *PEX10* expression in AR-positive C4-2 and LNCaP cells was significantly higher than in DU145 and PC-3 cells (Figs. 4B and S4A), suggesting that AR can upregulate *PEX10* expression.

To further confirm this hypothesis, we knocked down *AR* in C4-2 and LNCaP cells and detected PEX10 expression at the protein and mRNA levels. As expected, PEX10 expression at the protein and mRNA levels remarkably decreased after *AR* knockdown (Fig. 4C–E). We also overexpressed *AR* in C4-2 and LNCaP cells and confirmed an elevation of KLK3 and PEX10 expression in both mRNA and protein level (Fig. S4B–E). Furthermore, the knockdown of *AR* in C4-2 and LNCaP cells significantly increased H_2_O_2_ levels in prostate cancer cells (Fig. 4F, G) and led to a significant downregulation of PMP70 levels (Fig. 4H–J).

In addition to the TCGA database showing a significant positive correlation between the *AR* or *KLK3* expression with *PEX10* expression (Figs. 4K and S4F, G), we also found a considerable positive correlation between AR protein and PEX10 expression level by immunohistochemistry staining of tissue microarray from prostate cancer patients (*n* = 70, *P* < 0.0001) (Fig. 4L, M). Additionally, we noticed a positive correlation between the PEX10 expression level with Gleason scores (Fig. S4H), which indicated that PEX10 may be the downstream target of AR.

The ectopic expression of *PEX10* could reverse the downregulation of *PEX10* expression after *AR* knockdown (Figs. 4N and S4I), and the change of ROS and PMP70 levels in C4-2 and LNCaP cells induced by *AR* knockdown could also be reversed by the ectopic expression of PEX10 (Figs. 4O and S4J–L). These results indicate that AR exerts a significant positive regulation on the expression and function of PEX10 which could mediate AR regulation of prostate cancer cell oxidative stress.

### *PEX10* is an AR target gene and can be regulated by AR inhibition and activation

**Fig. 5:**
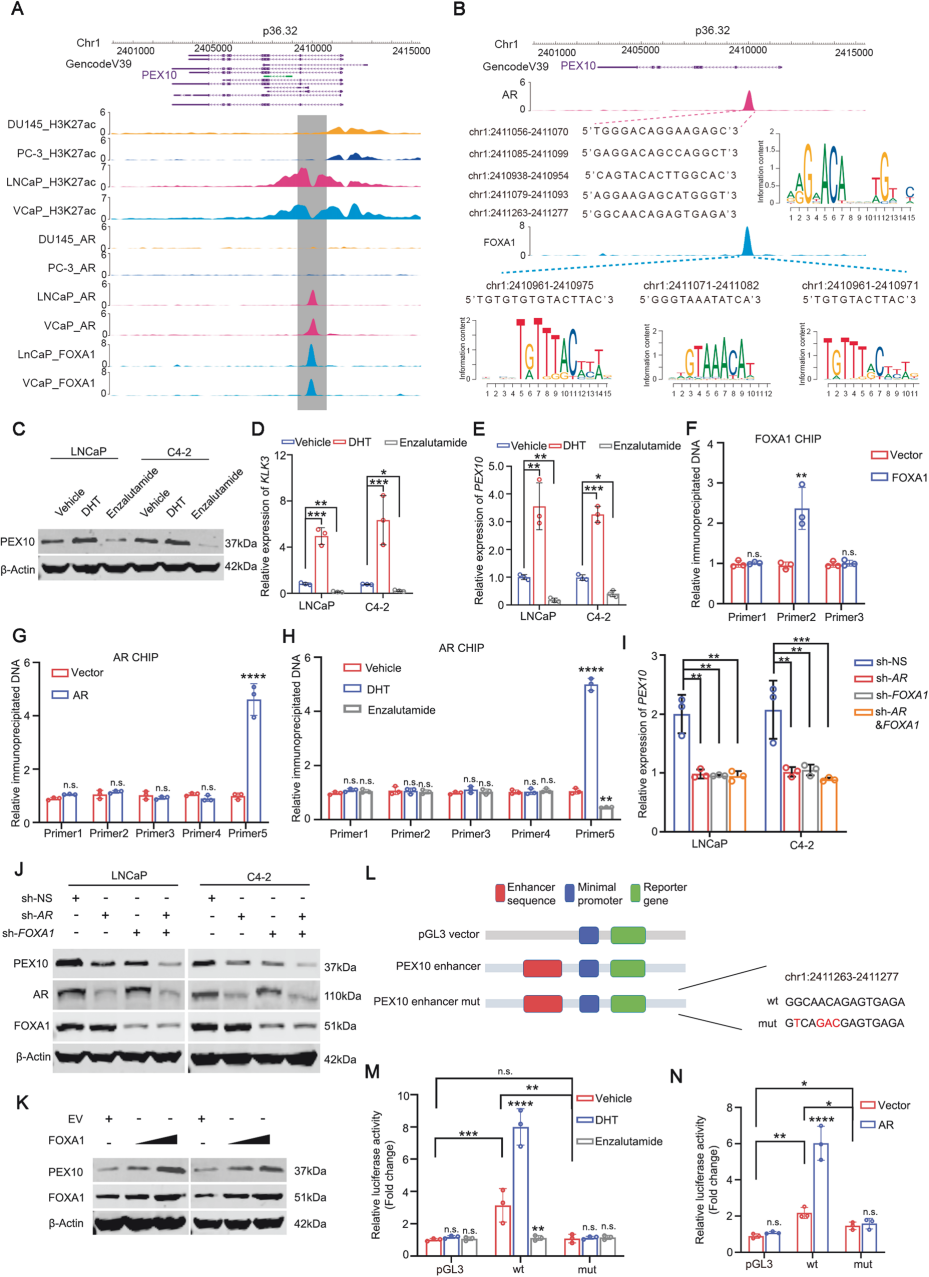
PEX10 is an AR target gene and can be regulated by AR inhibition and activation. **A** ChIP-seq binding peak of H3K27ac, AR, and FOXA1 on *PEX10* gene of prostate cancer cell lines (DU145, PC-3, VCaP, and LNCaP). **B** Lists of potential binding sequences of AR and FOXA1 in *PEX10* 2411280-2410760 region. **C** PEX10 expression at protein level in AR-positive prostate cancer cells (C4-2 and LNCaP) after treatment with DHT (100 nM) or enzalutamide (5 μM). **D**, **E** *KLK3* and *PEX10* expression at mRNA level in AR-positive prostate cancer cells (C4-2 and LNCaP) after treatment with DHT (100 nM) or enzalutamide (5 μM). Unpaired t-test. (*P* < 0.05 as “*”; *P* < 0.01 as “**”; *P* < 0.001 as “***”). **F**, **G** ChIP-qPCR showed the AR or FOXA1 binding change after AR or FOXA1 was ectopically expressed in C4-2 cells. **H** ChIP-qPCR showed the AR binding change after treatment with DHT (100 nM) or enzalutamide (5 μM). **I** The expression of *PEX10* at the mRNA level after AR or FOXA1 knockdown alone or in combination. **J** The expression of PEX10 at the protein level after AR or FOXA1 knockdown alone or in combination. **K** The expression of PEX10 with the supplementation and a different dose of FOXA1. **L** The diagram shows the construction of the wild type or mutant PEX10 enhancer plasmid. **M** The luciferase level after transfection with wild-type or mutant PEX10 enhancer plasmid and treated with DHT (100 nM) or enzalutamide (5 μM). Unpaired t-test. (n.s. no specific; *P* < 0.01 as “**”; *P* < 0.001 as “***”, *P* < 0.0001 as “****”). **N** The luciferase level after transfection with wild-type or mutant PEX10 enhancer plasmid after ectopic expression of AR. Unpaired t-test. (n.s. no specific, *P* < 0.05 as “*”; *P* < 0.01 as “**”, *P* < 0.0001 as “****”).

As AR usually functions as a transcription factor, we explored whether *PEX10* is the direct target gene of AR and re-analyzed the AR ChIP-seq results from public chip-seq datasets in the Cistrome database (http://cistrome.org/db). In both the VCaP and LNCaP cells, we identified a notable binding peak in the 2411280-2410760 region of the first intron of the *PEX10* gene (Fig. 5A). We also detected a remarkable increase in H3K27 acetylation level in this region in both VCaP and LNCaP cells (Fig. 5A). Conversely, in DU145 and PC-3 cells, we did not detect any notable AR binding in the 2411280-2410760 region or whole *PEX10* gene, and the H3K27 acetylation level did not change significantly (Fig. 5A). This result suggests that the AR may directly bind to the *PEX10* 2411280-2410760 region and regulate the expression of *PEX10*. Coincidentally, we found that FOXA1 also showed notable binding in the 2411280-2410760 region of the *PEX10* gene in VCaP and LNCaP cells (Fig. 5A). These results suggest that there may be an enhancer in the *PEX10* 2411280-2410760 region, and AR and FOXA1 cooperate to activate this enhancer and promote the expression of *PEX10*. AR and FOXA1 frequently co-regulate gene expression in prostate cancer [10, 35].

In light of this, we conducted further PPI analysis of AR in three databases, revealing its interaction with FOXA1 (Fig. S5A–C). Additionally, our Co-IP results further confirm FOXA1’s binding with AR in prostate cancer (Fig. S5D). Subsequent analysis of TCGA data showed that besides AR, FOXA1 also exhibits a significant positive correlation with PEX10 (Fig. S5E). We then stratified prostate cancer into FOXA1 high-expression and low-expression groups using a cut-off of 7.5. Further analysis indicated a positive correlation between AR and PEX10 in the FOXA1 high-expression group, while this correlation was not statistically significant in the FOXA1 low-expression group (Fig. S5E). These findings suggest that AR’s regulation of PEX10 expression depends on the presence of FOXA1.We identified five motifs with high binding potential (JASPAR score ≥ 90) in combination with the prediction results of the JASPAR database. Consistently, we identified three high-scoring potential FOXA1 binding motifs in this region (Fig. 5B).

Dihydrotestosterone (DHT) treatment significantly increased *PEX10* expression at the mRNA and protein levels in prostate cancer cells, including C4-2 and LNCaP cells, while enzalutamide treatment decreased *PEX10* expression, which is consistent with the changes in *KLK3* (Fig. 5C–E) and our previous conclusions. To further identify the binding positions of AR and FOXA1 on the *PEX10* gene, we constructed corresponding primers and used ChIP-qPCR to identify the binding potential of AR and FOXA1. The binding of FOXA1 to FOXA1-Primer2, but not to other primers, significantly increased after ectopic expression of FOXA1 in C4-2 cells (Fig. 5F), confirming that Primer2 is more likely to be where FOXA1 binds to the *PEX10* gene. Similarly, after ectopic expression of AR in C4-2 cells, the binding of AR-Primer5 to AR was remarkably enhanced, whereas the binding levels of other primers to AR did not change significantly (Fig. 5G). Enhanced AR-Primer5 binding was also observed in C4-2 cells after treatment with DHT, while decreased binding of AR-Primer5 was observed after enzalutamide treatment (Fig. 5H). These results further confirmed that the AR-Primer5 region is where AR bind to the *PEX10* gene.

We wanted to determine whether AR and FOXA1 were involved in the classic mode of *PEX10* regulation. We individually knocked down *FOXA1* and *AR* in LNCaP and C4-2 cells, respectively, which downregulated the expression of *PEX10*; however, their combined knockdown did reduce the expression of *PEX10* further (Fig. 5I, J). Consistently, FOXA1 increased the expression of *PEX10* in a gradient-dependent manner (Fig. 5K). These results show that FOXA1 and AR are involved in the same transcriptional regulation pathway for *PEX10*, but do not regulate *PEX10* expression separately.

To further explore the role of the enhancer sequence in *PEX10*, we constructed an enhancer plasmid containing the sequence, containing a minimal promoter and a fluorescently expressed gene that could not be activated under normal conditions (Fig. 5L). We did not observe significant fluorescence in plasmids that did not contain this enhancer sequence, whereas we observed notable fluorescence after adding this enhancer sequence upstream of the promoter (Fig. 5M, N). Ectopic expression of AR significantly enhanced the fluorescence levels (Fig. 5M, N). Additionally, DHT and enzalutamide treatment significantly increased and decreased fluorescence, respectively (Fig. 5M, N). This result confirms the role of this enhancer and its activation by AR. When we mutated the AR binding motif, ectopic AR expression or DHT treatment no longer enhanced fluorescence, and enzalutamide did not inhibit fluorescence (Fig. 5M, N). Additionally, we performed luciferase assays targeting FOXA1, further confirming its ability to bind to and enhance the activity of *PEX10* enhancers (Fig. S5F).

This finding further confirmed that AR/FOXA1 could target the *PEX10* enhancer and activate *PEX10* transcription, consistent with our previous hypothesis (Fig. S5G).

### Enzalutamide sensitize prostate cancer cells to ferroptosis inducers

**Fig. 6:**
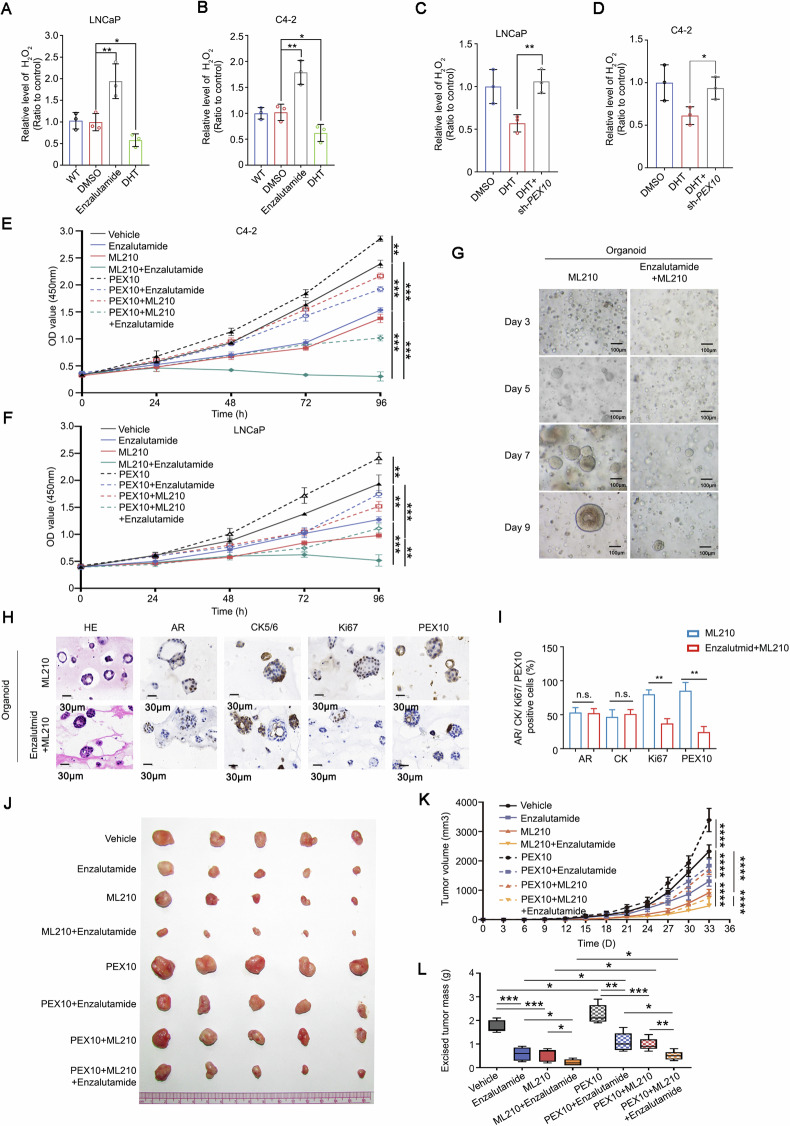
Enzalutamide sensitize prostate cancer cells to ferroptosis inducers. **A**, **B** H_2_O_2_ level after treatment with vehicle, DHT (100 nM) or enzalutamide (5 μM) in prostate cancer cell lines (C4-2 and LNCaP). Unpaired t-test. (*P* < 0.05 as “*”; *P* < 0.01 as “**”). **C**, **D** H_2_O_2_ level after treatment with DHT and then knocked down *PEX10* in prostate cancer cell lines (C4-2 and LNCaP). Unpaired t-test. (*P* < 0.05 as “*”; *P* < 0.01 as “**”). **E**, **F** The CCK-8 OD value of two *PEX10* overexpression or overexpression control prostate cancer cell lines (C4-2 and LNCaP) after treatment with ML210 (2 μM) and combination with or without enzalutamide (5 μM). ANOVA. (*P* < 0.01 as “**”; *P* < 0.001 as “***”). **G** The growth state of patient prostate cancer tissue original organoid after treatment with ML210 (2 μM) or enzalutamide (5 μM) combination from days 1 to 9. Expression (**H**) and statistic (**I**) of AR, CK5/6, Ki67 and PEX10 in organoids of different groups by IHC method. Unpaired t-test. (n.s. no specific; *P* < 0.01 as “**”). **J**–**L** The image representative of in vivo tumor xenograft model (C4-2-R) (**J**) performed on SCID mice (*n* = 5) and the volume (**L**) and weight (**K**) statistic of the tumor with the constant treatment of ML210 (5 mg/kg) and/or enzalutamide (10 mg/kg), ANOVA. (*P* < 0.0001 as “****”). Unpaired t-test. (*P* < 0.05 as “*”; *P* < 0.01 as “**”).

Our results suggest that ML210 promote *PEX10* expression and lead to the elimination of ROS in prostate cancer, while enzalutamide could inhibit PEX10 function and synergy with ML210 to exert a better anti-tumor effect. To validate this hypothesis, we assessed the changes in H_2_O_2_ levels in C4-2 and LNCaP cells following treatment with DHT and enzalutamide, respectively. DHT exhibited a notable reduction in H_2_O_2_ levels, while enzalutamide led to an elevation of H_2_O_2_ levels in prostate cancer cells (Fig. 6A, B). Importantly, in the absence of PEX10, DHT no longer decreased H_2_O_2_ levels, confirming that enzalutamide could mitigate ROS in prostate cancer by inhibiting *PEX10* (Fig. 6C, D).

We supplemented our study with MDA experiments (Fig. S6A), demonstrating that enzalutamide indeed promotes lipid peroxidation and ferroptosis in prostate cancer. Additionally, Western blot results showed no significant changes in necroptosis of prostate cancer cells (Fig. S6B). Research has indicated that AR antagonists sensitize AR^+^ prostate cancer to ferroptosis by downregulating MBOAT2 [36]. Therefore, we conducted WB validation for GPX4 and MBOAT2 simultaneously and found that AR indeed enhances MBOAT2 rather than GPX4 in prostate cancer (Fig. S6C). Additionally, we found that overexpression of PEX10 reduces lipid peroxidation and ferroptosis in prostate cancer (Fig. S6D). Moreover, the overexpression of PEX10 does not directly affect GPX4 expression (Fig. S6E). These results suggest that the promotion of ferroptosis via the AR/FOXA1 − PEX10 axis operates independently of GPX4.

On the other hand, we treated *PEX10* overexpression or control cells with ML210, enzalutamide, or a combination of both. The results showed that both ML210 and enzalutamide treatment could inhibit the proliferation of prostate cancer cells, and their combined treatment could achieve a better inhibitory effect on prostate cancer cell growth. Contrarily, *PEX10* overexpression partially abolished the tumor cell inhibitory effects of both ML210 and enzalutamide (Fig. 6E, F). To demonstrate that inhibiting PEX10 on this basis could sensitize enzalutamide or enhance the effect of combining ML210 with enzalutamide, we supplemented our study with IC50, CCK8, and colony formation assays (Fig. S7A–C). The IC50 results indicate that reducing PEX10 increases the sensitivity of prostate cancer to enzalutamide (Fig. S7C). Additionally, both the CCK8 and colony formation assay results reveal that the combined effect of ML210 and enzalutamide is enhanced upon downregulation of PEX10 (Fig. S7A, B). This finding demonstrates the critical role of PEX10 in regulation of ML210 and enzalutamide sensitivity in prostate cancer. Otherwise, the combination of enzalutamide and ML210 inhibited the growth of organoids derived from human CRPC tissue when compared with enzalutamide (5 μM, 9 days) treatment alone (Figs. 6G and S7D). Additionally, ML210 and enzalutamide combination showed to downregulate PEX10 expression and downregulate Ki67 level in CRPC organoids (Figs. 6H, I and S7E). We examined the anti-tumor effects of ML210 and enzalutamide to C4-2 in vivo in SCID mice. Results showed ML210 and enzalutamide combination administration could significantly inhibit the tumor growth, and both the xenograft size and tumor growth rate significantly increased after *PEX10* overexpression (Fig. 6J–L). Meanwhile, ML210 and enzalutamide co-treatment in vivo in SCID mice could more effectively inhibit tumor growth, Ki67 and induce Cleaved-Caspase-3 expression (Fig. S8A, B), and reactivate H_2_O_2_ level (Fig. S8C). Additionally, we developed the enzalutamide-resistant prostate cancer cell line C4-2R (Fig. S9A, B). The combination of ML210 and enzalutamide demonstrated highly effective anticancer activity in these resistant cells (Fig. S9C). Additionally, Additionally, PEX10 knockdown further enhanced their anticancer effects (Fig. S9C). This suggests that the combination of ML210 and enzalutamide not only performs better in regular prostate cancer but also shows promising results in enzalutamide-resistant cells. Additionally, we demonstrated that knocking down PEX10 in enzalutamide-resistant cells can restore their sensitivity to enzalutamide (Fig. S9D).

All the results demonstrate the strong potential of the combination of ML210 and enzalutamide in preventing prostate cancer.

## Discussion

**Fig. 7:**
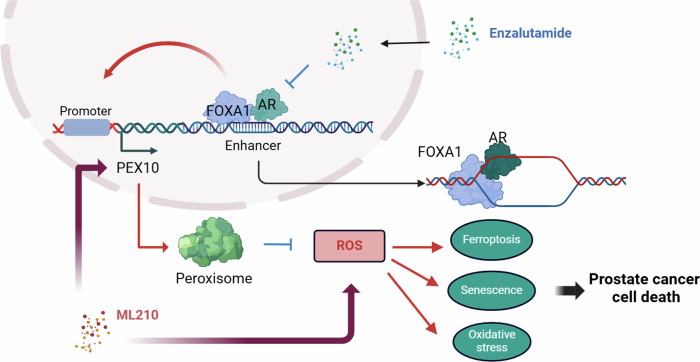
Hypothetical model depicting the mechanistic Enzalutamide activates prostate cancer cells to ML210. AR and FOXA1 bind the *PEX10* enhancer and promote *PEX10* promoter function. PEX10 participates in peroxisome formation and ROS process and finally inhibits the ROS. Enzalutamide could inhibit AR function and finally promote ROS. Enzalutamide could activate the ROS in prostate cancer cells and sensitize cells to ML210. Created with BioRender.com (www.biorender.com).

AR play a pivotal role in orchestrating the regulatory mechanisms governing the normal functioning of the prostate, as well as influencing conditions such as benign prostatic hyperplasia and prostate cancer [37–39]. It is widely posited that AR serves as the pivotal intermediary in the prostate’s response to androgens [39]. As an androgen-activated receptor, AR has a conserved structural and functional organization, which could activate its downstream target genes such as KLK3 and TMPRESS2 and pathways to elicit pro-tumorigenesis and development effect [40]. Here, we revealed that *PEX10* as a new AR downstream target and could be activated by AR at the transcriptional level. In addition, as a downstream gene of the AR, *PEX10* may involve in the ROS depletion, ferroptosis sensitivity and cellular senescence of prostate cancer and promote prostate cancer cell proliferation. In somehow, the AR-*PEX10* pathway therefor inhibit the ROS accumulation in prostate cancer cells (Fig. 7).

Therapy based on inhibition of androgen and androgen receptor has always been the first-line treatment of prostate cancer. In addition, in recent years, many non-targeted AR pathway therapies, such as PARP inhibitor or immune therapy [41–43], have been explored and discovered, aiming to explore new ways of prostate cancer treatment and combination drugs to enhance the therapeutic effect. The discovery of enzalutamide was a breakthrough in AR antagonist therapy [9, 44]. In addition to its classic AR pathway inhibition, enzalutamide has also been found to exert a mechanism of prostate cancer inhibition through non-AR inhibition [45] or combine treatment [46, 47], which further strengthens its position as a cornerstone of prostate cancer treatment [48]. Within our research, enzalutamide demonstrated the capacity to impede the expression of PEX10 by suppressing the AR-*PEX10* pathway. This inhibition effectively obstructed the ROS inhibitory bypass, a consequence of PEX10 activation induced by ROS inducers. Consequently, intracellular ROS levels were elevated, culminating in the ultimate suppression of prostate cancer progression (Fig. 7). This study offers theoretical underpinning for advocating the combined application of enzalutamide, thereby broadening its clinical indications in the context of prostate cancer.

ROS represent a class of exceptionally reactive oxidative molecules, including entities such as superoxide ions, hydrogen peroxide, and various free radicals, among others. They play a pivotal role in numerous fundamental cellular biological processes [49]. Certain scholars posit that in the context of prostate cancer, the levels of ROS could be correlated with the survival and proliferation of cancer cells. On one hand, moderate levels of ROS might engage in the regulation of signaling pathways [50, 51], prompting normal cells to sustain their physiological state. Conversely, excessively elevated or diminished levels of ROS could instigate intracellular oxidative stress, consequently impacting normal cellular functions [52, 53]. PEX10 is a protein intricately involved in the formation and upkeep of the peroxisomal membrane, playing a pivotal role in ensuring the functionality of peroxisomes. Simultaneously, peroxisomes, as cellular organelles, actively engage in the metabolism and regulation of ROS, contributing to the preservation of ROS balance within the cell [54]. In general, tumor cells commonly employ a diverse array of mechanisms to mitigate the deleterious effects arising from the production of ROS [55]. Our research indicates that in prostate cancer, the heightened expression of AR achieves this goal by facilitating the expression of *PEX10*, a process that can be suppressed by enzalutamide. The mechanism may help us understand the ROS function and regulation in prostate cancer.

As delineated in this study, *PEX10* appears to exert an influence on the trajectory of prostate cancer by instigating oxidative stress, ferroptosis, and cellular senescence. Notably, the mechanism of ferroptosis in prostate cancer has been comprehensively examined. Although the effects of AR and enzalutamide on ferroptosis in prostate cancer were observed in our study, the relationship between AR, enzalutamide, and ferroptosis remains unclear. Our ongoing research (not shown here) and other studies [36, 56] have shown that enzalutamide may also affect lipid oxidation in prostate cancer and exert an effect on ferroptosis in prostate cancer. Additionally, ferroptosis inducers may affect many other physiological functions in the human body, leading to uncertain side effects. Nevertheless, there is still a need for solid basic and clinical research before practical clinical applications. Exploring suitable biomarkers for the further development of in vivo studies and clinical monitoring is also necessary. Further studies are needed to elucidate the mechanism of ferroptosis in enzalutamide resistance.

In summary, our study demonstrates a previously unrecognized function of AR inhibiting ROS accumulation by upregulating PEX10 and suggests a new strategy of using enzalutamide in prostate cancer treatment.

### Supplementary information

Supplementary material

Original western blots

## Data Availability

The datasets used and/or analyzed during the current study are available from the corresponding author on reasonable request.
