# Supplementary material for: Enzalutamide inhibits PEX10 function and sensitizes prostate cancer cells to ROS activators
Source: Cell Death Dis. 2024 Aug 3;15(8):559. doi: 10.1038/s41419-024-06937-7 (PMC11297951; doi:10.1038/s41419-024-06937-7)
Supplement: Supplementary file 1 — Supplementary material [file 41419_2024_6937_MOESM1_ESM.pdf]

Supplementary Information

Enzalutamide inhibits PEX10 function and sensitizes prostate cancer cells to ROS activators

Yuankang Feng, Yu Zhang, Hao Li, Tao Wang, Fubo Lu, Ruoyang Liu, Guoqing Xie, Liang Song, Budeng Huang, Xiang Li, Yinghui Ding, Jinjian Yang, Zhankui Jia, Zhenlin Huang

Files included in supplementary information

Supplementary Materials and methods

Supplementary Figure 1 （Supplemental to Figure 1） PEX10 is involved in ROS in prostate cancer.

Supplementary Figure 2 （Supplemental to Figure 2） PEX10 suppresses cell death by decreasing ROS and H_2O_2.

Supplementary Figure 3 （Supplemental to Figure 3） Knocking down PEX10 enhances the anticancer efficacy of ML210.

Supplementary Figure 4 （Supplemental to Figure 4） AR plays a critical role in regulation of PEX10 expression and function.

Supplementary Figure 5 （Supplemental to Figure 5） PEX10 is an AR target gene and can be regulated by AR inhibition and activation.

Supplementary Figure 6 （Supplemental to Figure 6） Enzalutamide promotes ferroptosis through a GPX4-independent pathway.

Supplementary Figure 7 （Supplemental to Figure 6） Enzalutamide sensitize prostate cancer cells to ferroptosis inducers.

Supplementary Figure 8 （Supplemental to Figure 6） Enzalutamide sensitizes prostate cancer cells to ferroptosis inducers in vivo.

Supplementary Figure 9 （Supplemental to Figure 6） Sh-*PEX10* re-sensitive prostate cancer cells to Enzalutamide.

Supplementary Table S1. Cell lines, antibodies and other reagents and resources

Supplementary Table S2. Sequence information of shRNAs

Supplementary Table S3. Sequence information of primers for RT-qPCR

Supplementary Materials and methods

Tissue specimens

Ten pairs of tissue samples were collected from prostate cancer patients between 2023 and 2024 at the First Affiliated Hospital of Zhengzhou University (Zhengzhou, China). The patients in this study received a definite diagnosis by histopathological examination and had not undergone medical treatment. Written informed consent was obtained from every patient included in the study. The research was approved by the Research Ethics Committee of the First Affiliated Hospital of Zhengzhou University (2022-KY-0239-002).

Colony formation assay

The proliferative ability of the cells was analyzed using a colony formation assay. Two milliliters of 1640 medium containing 10% FBS and 3000 cells were added to each well of a 6-well plate. The cells were cultured for 12 days and fixed with paraformaldehyde for 30 min. The cell colonies were then stained with crystal violet staining solution for 2 h. A microscope (Leica DMIRB, Wetzlar, Germany) was used to capture images.

CCK8 assay

Cells were plated in 96-well plates at 3×10^3 cells/well. 10 microliter(mL)s of CCK-8 reagent (Dojindo Molecular Technologies, Inc., Rockville, MD, USA) were added to each well and incubated for 1 h at the indicated time points (0, 1-, 2-, 3-, and 4-days post-transfection). Optical density at 450 nm (OD450) was measured using a microplate reader.

Reverse transcription-quantitative polymerase chain reaction (RT-qPCR)

The extracted total RNA was incubated with gDNA remover at 36°C for 30 min to remove genomic DNA. cDNA was synthesized using a cDNA synthesis kit (Takara Bio Inc., Kusatsu, Japan), following the manufacturer's instructions. SYBR Green Mix (Roche Diagnostics, Basel, Swiss Confederation) and a QuantStudio 3 Real-Time PCR System (Thermo Fisher Scientific, Inc., Waltham, MA, USA) were used to perform qPCR according to the manufacturers' protocol. The reaction conditions of the 20 μL system were: the first reaction at 95°C for 30 s, and then a 95°C reaction for 10 s, 60°C reaction for 30 s, and 72°C reaction for 10 s, repeated 45 times. The relative expression levels of the indicated genes were quantified using the $2^{-\Delta\Delta ct}$ method. GAPDH was used as an internal control (Primers information in Supplementary Table 3).

Immunofluorescence staining

We dropped 6×10^5 cells onto round coverslips to make cell coverslips. The prepared cell coverslips were soaked in paraformaldehyde for 10 min, then washed with distilled water for 5 min. The coverslips were incubated in a buffer containing 0.5% Triton X-100 reagent at room temperature for 20 min. The samples were then washed with PBS (3 min × 3) and sealed with goat serum at room temperature for 30 min. Then, diluted primary antibodies were added and incubated overnight at 4°C. After washing thrice, the corresponding secondary antibody was added and incubated for approximately 1 h. Phenyl indole (DAPI) reagent was added, and the slices were incubated in the dark for 5 min. After washing, the slides were fixed with an anti-fluorescence quenching agent and observed under a fluorescence microscope. We conducted three independent experiments.

MDA assay

For *in vitro* experiment, MDA staining was performed using the MDA Kit (Beyotime, Shanghai, China) according to the manufacturer’s instructions.

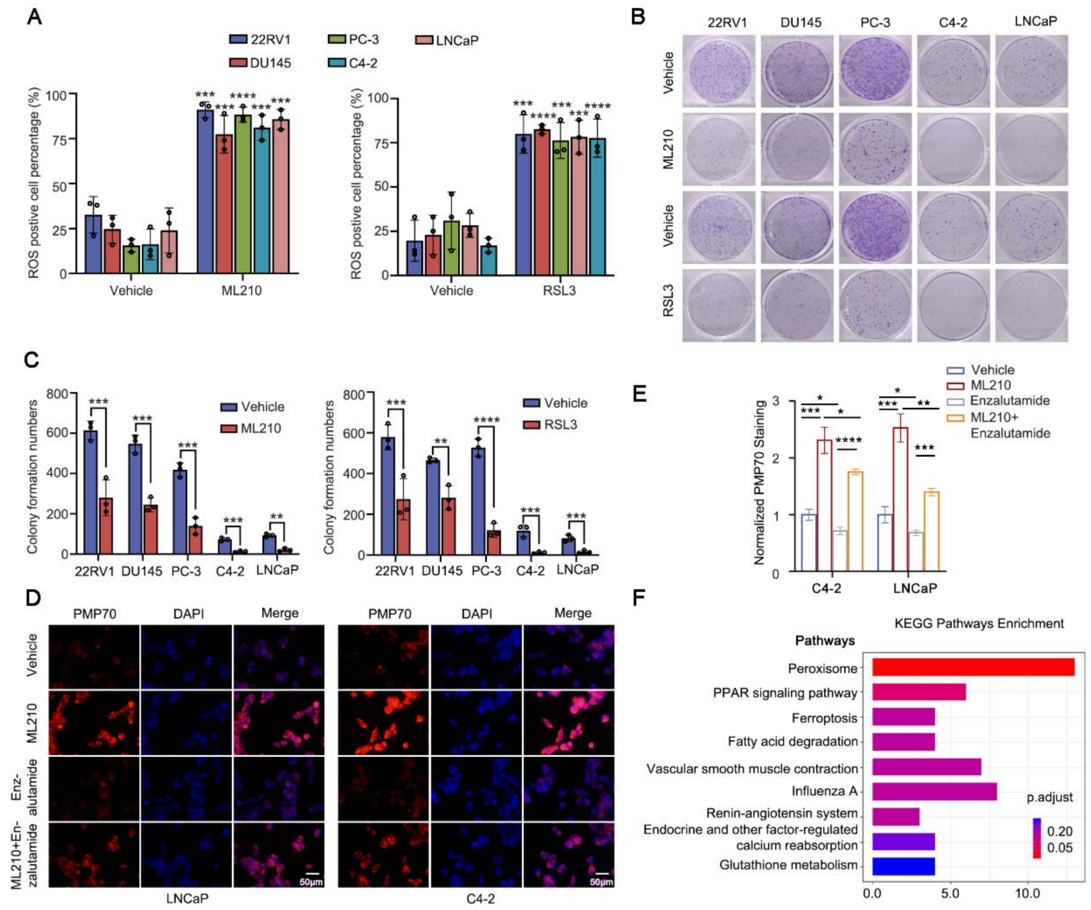

Supplementary Figure 1, Supplemental to Figure 1

A, The statistic of ROS level of prostate cancer cell lines (22Rv1, DU145, PC-3, C4-2 and LNCaP) after treat with ML210 or RSL3. Unpaired t test. (*P*< 0.001 as “***”; *P*< 0.0001 as “****”). **B** and **C,** The cell colony and statistic of prostate cancer cell lines (22Rv1, DU145, PC-3, C4-2 and LNCaP) after treat with ML210 or RSL3. Unpaired t test. (*P* < 0.01 as “**”; *P*< 0.001 as “***”; *P*< 0.0001 as “****”). **D** and **E,** PMP70 expression level in prostate cancer cells (C4-2 and LNCaP) after treat with ML210 (2μM) or Enzalutamide (5μM). Unpaired t-test. (*P* < 0.05 as “*”; *P* < 0.01 as “**”, *P* < 0.001 as “***” , *P* < 0.0001 as “****”). **F,** The images were the representative of the pathways with the most significance changed pathway after ML210treatment from KEGG analysis.

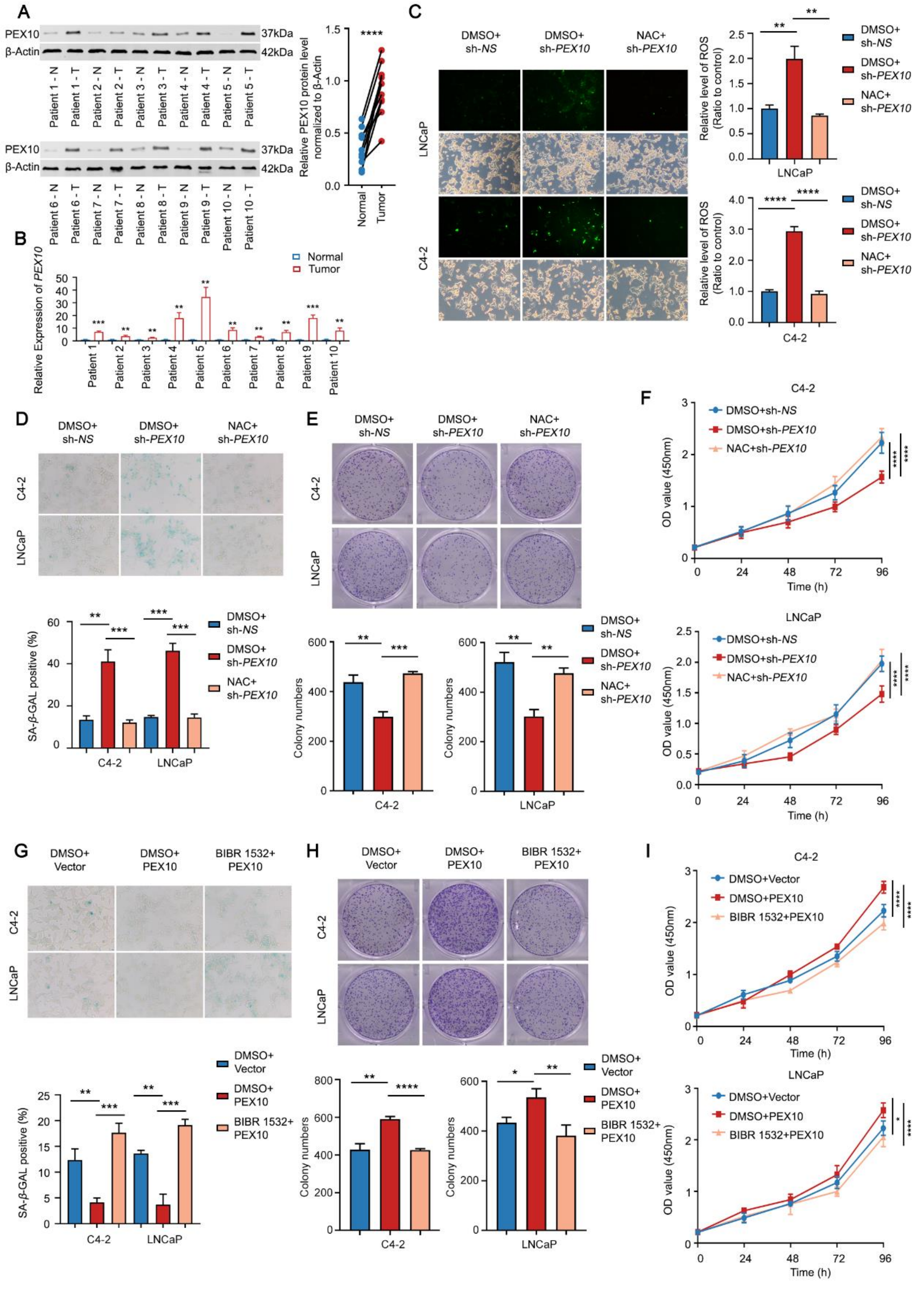

Supplementary Figure 2, Supplemental to Figure 2

A-B, RT-qPCR (**B**) and Western blot analysis (**A**) showed the PEX10 expression in prostate cancer and normal tissues. β-Actin served as internal references. ($P < 0.01$ as “**”; $P < 0.001$

as “***”, *P* < 0.0001 as “****”). **C,** ROS level in prostate cancer cells (C4-2 and LNCaP) after *PEX10* knockdown alone or in combination with NAC (5 mM). Unpaired t-test. (*P* < 0.01 as “**”, *P* < 0.0001 as “****”). **D,** SA-β-GAL level in prostate cancer cells (C4-2 and LNCaP) after *PEX10* knockdown alone or in combination with NAC (5 mM). Unpaired t-test. (*P* < 0.01 as “**”, *P* < 0.001 as “***”). **E,** Colony numbers and statistic in prostate cancer cells (C4-2 and LNCaP) after *PEX10* knockdown alone or in combination with NAC (5 mM). Unpaired t-test. (*P* < 0.01 as “**”, *P* < 0.001 as “***”) **F,** CCK8 OD value and statistic in prostate cancer cells (C4-2 and LNCaP) after *PEX10* knockdown alone or in combination with NAC (5 mM). Unpaired t-test. (*P* < 0.0001 as “****”). **G,** SA-β-GAL level in prostate cancer cells (C4-2 and LNCaP) after overexpression of *PEX10* alone or in combination with BIBR 1532 (40 μM). Unpaired t-test. (*P* < 0.01 as “**”, *P* < 0.001 as “***”). **H,** Colony numbers and statistic in prostate cancer cells (C4-2 and LNCaP) after overexpression of *PEX10* alone or in combination with BIBR 1532 (40 μM). Unpaired t-test. (*P* < 0.05 as “*”, *P* < 0.01 as “**”, *P* < 0.0001 as “****”). **I,** CCK8 OD value and statistic in prostate cancer cells (C4-2 and LNCaP) after overexpression of *PEX10* alone or in combination with BIBR 1532 (40 μM). Unpaired t-test. (*P* < 0.05 as “*”, *P* < 0.0001 as “****”).

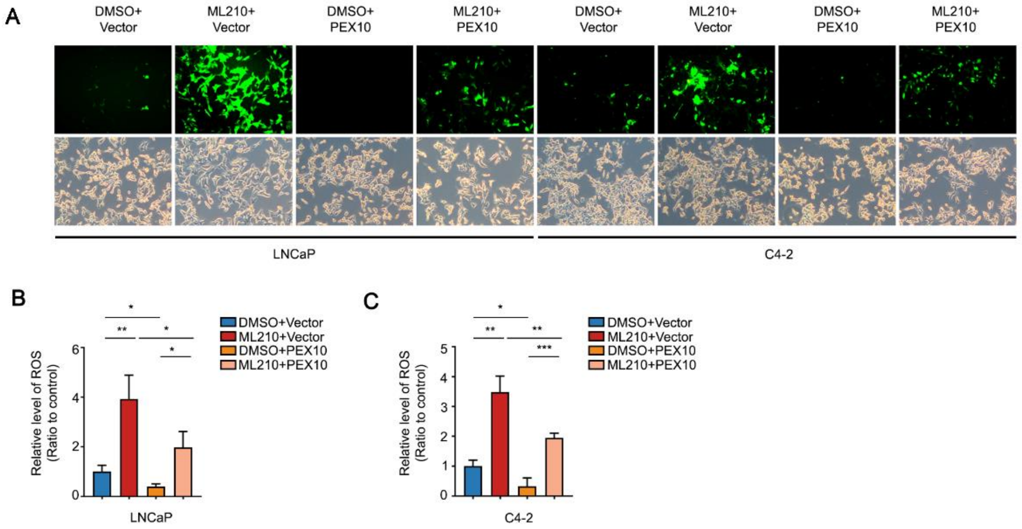

Supplementary Figure 3, Supplemental to Figure 3.

A-C, ROS level in prostate cancer cells (C4-2 and LNCaP) after treating with ML210 (2 μM) and (or) overexpression of *PEX10*. Unpaired t-test. (P < 0.05 as "*"; P < 0.01 as "**", P < 0.001 as "***").

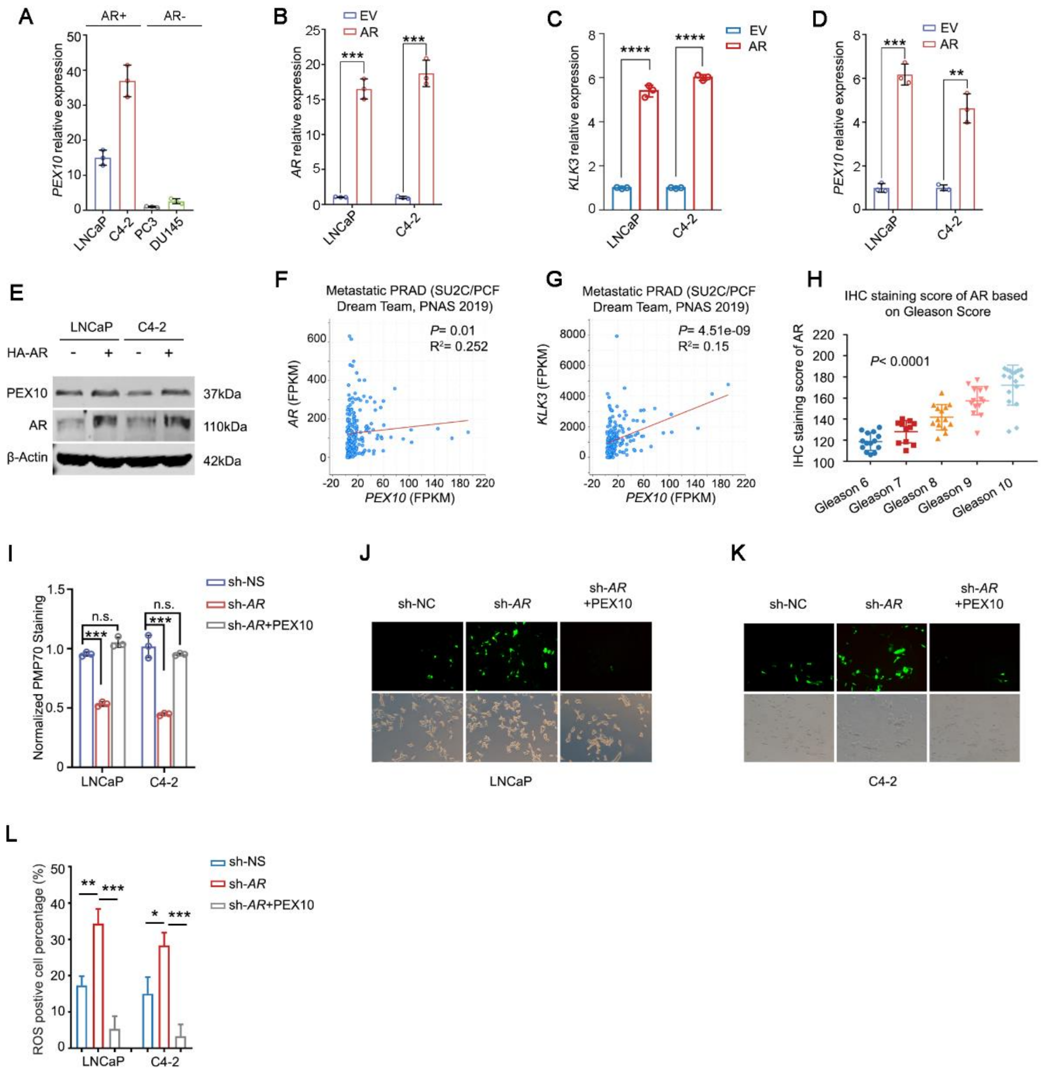

Supplementary Figure 4, Supplemental to Figure 4.

A, The expression of *PEX10* in AR-positive prostate cancer cells and in AR-negative prostate cancer cells. **B-D,** The expression of *AR*, *KLK3* and *PEX10* at the mRNA level in prostate cancer cells (C4-2 and LNCaP) after *AR* ectopically expressed. Unpaired t-test. ($P < 0.01$ as "**"; $P < 0.001$ as "***"; $P < 0.0001$ as "****"). **E,** The expression of AR and PEX10 at the protein level in prostate cancer cells (C4-2 and LNCaP) after *AR* ectopically expressed. **F** and **G,** The image showed the expression correlation of *AR* and *KLK3* with *PEX10* from TCGA cohort (Metastatic PRAD, SU2C/PCF Dream Team, PNAS 2019). **H,** The image showed the

IHC staining score of AR based on Gleason Score. One-way ANOVA. **I,** The statistic of the expression of PMP70 after *AR* knockdown alone or in combination with ectopically expressed of *PEX10* in C4-2 and LNCaP. Unpaired t-test. (n.s. as no specific, $P < 0.01$ as “**”; $P < 0.001$ as “***”). **J-L,** ROS level in prostate cancer cells (C4-2 and LNCaP) after *AR* knockdown alone or in combination with ectopically expressed of *PEX10*. Unpaired t-test. ($P < 0.05$ as “*”; $P < 0.01$ as “**”, $P < 0.001$ as “***”).

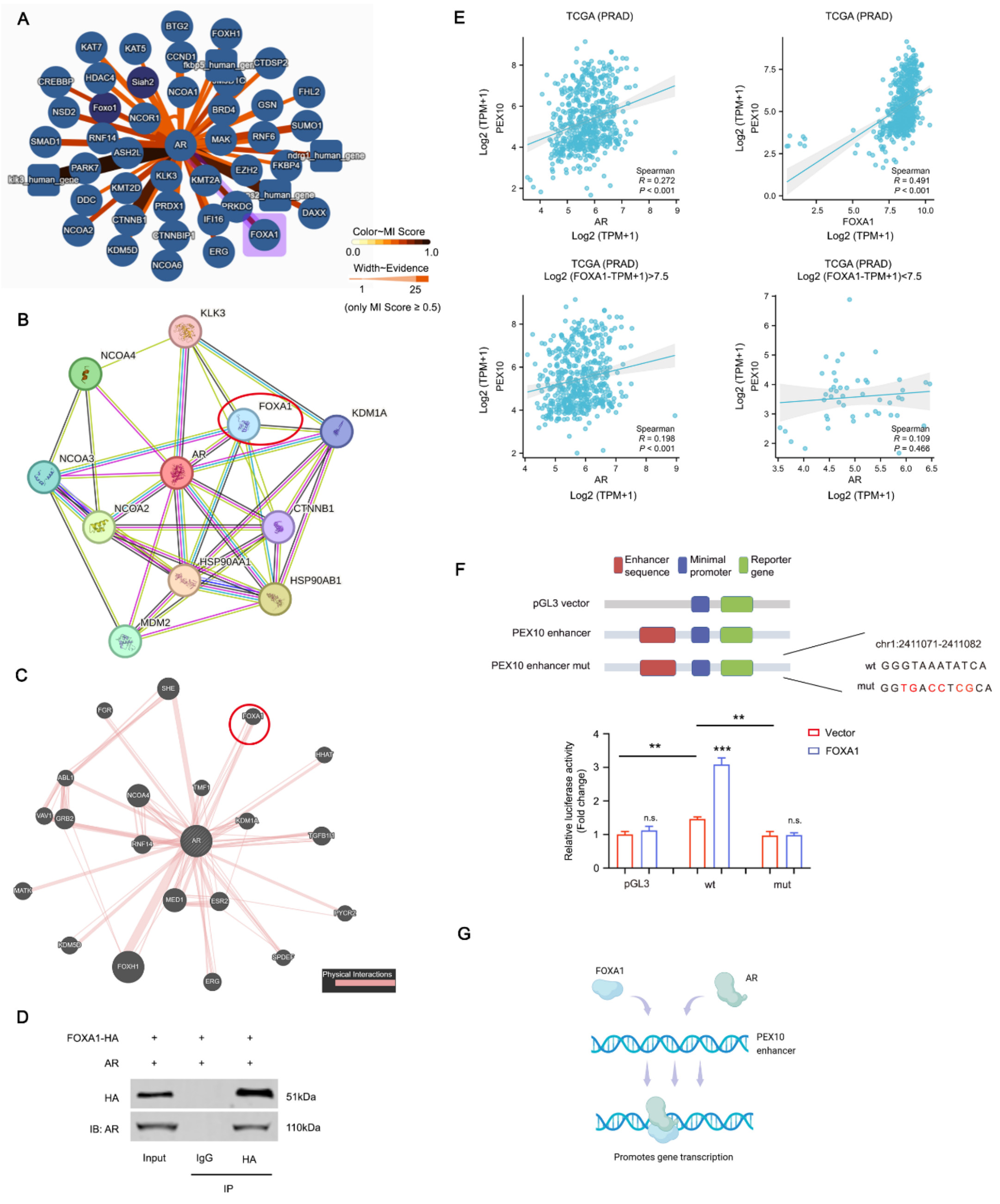

Supplementary Figure 5, Supplemental to Figure 5.

A-C, Proteins that can interact with AR were analyzed through the IntAct (A), STRING (B), and GENEMANIA (C) databases. **D,** Co-IP results showed the binding of AR to FOXA1 in prostate cancer cells C4-2. **E,** Correlation analysis of *PEX10* with *AR* and *FOXA1* from the TCGA database. **F,** The luciferase level after transfection with wild-type or mutant PEX10 enhancer plasmid after ectopic expression of *FOXA1*. Unpaired t-test. (n.s. no specific, $P <$

0.01 as “**”, $P < 0.001$ as “***”). **G,** The hypothetical model of AR and FOXA1 regulation of PEX10.

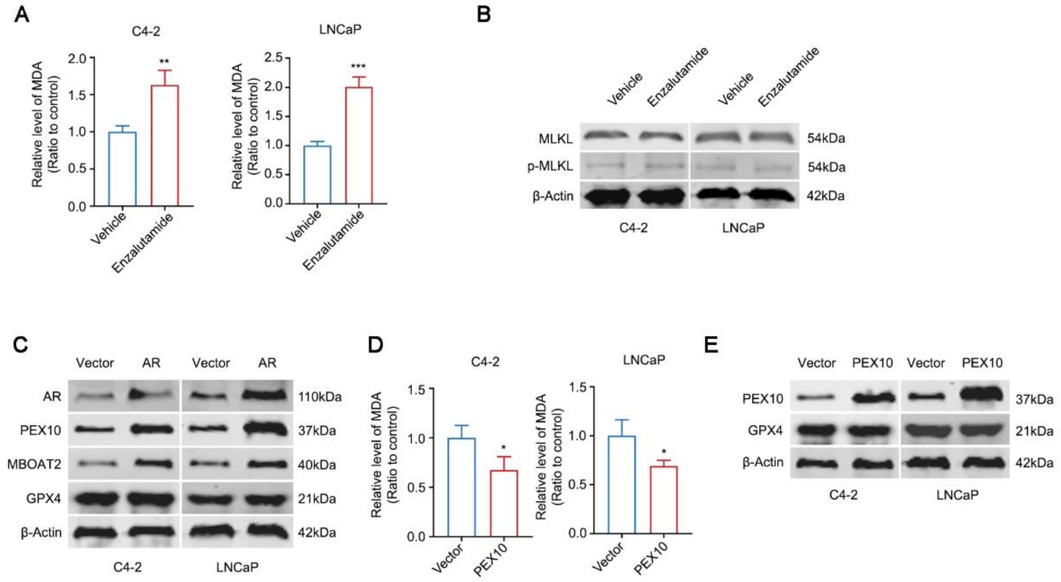

Supplementary Figure 6, Supplemental to Figure 6

A, MDA level in prostate cancer cells (C4-2 and LNCaP) after treating with enzalutamide (5μM). Unpaired t-test. (*P* < 0.01 as "**", *P* < 0.001 as "***"). **B,** The expression of MLKL and p-MLKL at the protein level in prostate cancer cells (C4-2 and LNCaP) after treating with enzalutamide (5μM). **C,** The expression of PEX10, MBOAT2, and GPX4 at the protein level in prostate cancer cells (C4-2 and LNCaP) after *AR* ectopically expressed. **D,** MDA level in prostate cancer cells (C4-2 and LNCaP) after *PEX10* ectopically expressed. Unpaired t-test. (*P* < 0.05 as "*"). **E,** The expression of GPX4 at the protein level in prostate cancer cells (C4-2 and LNCaP) after *PEX10* ectopically expressed.

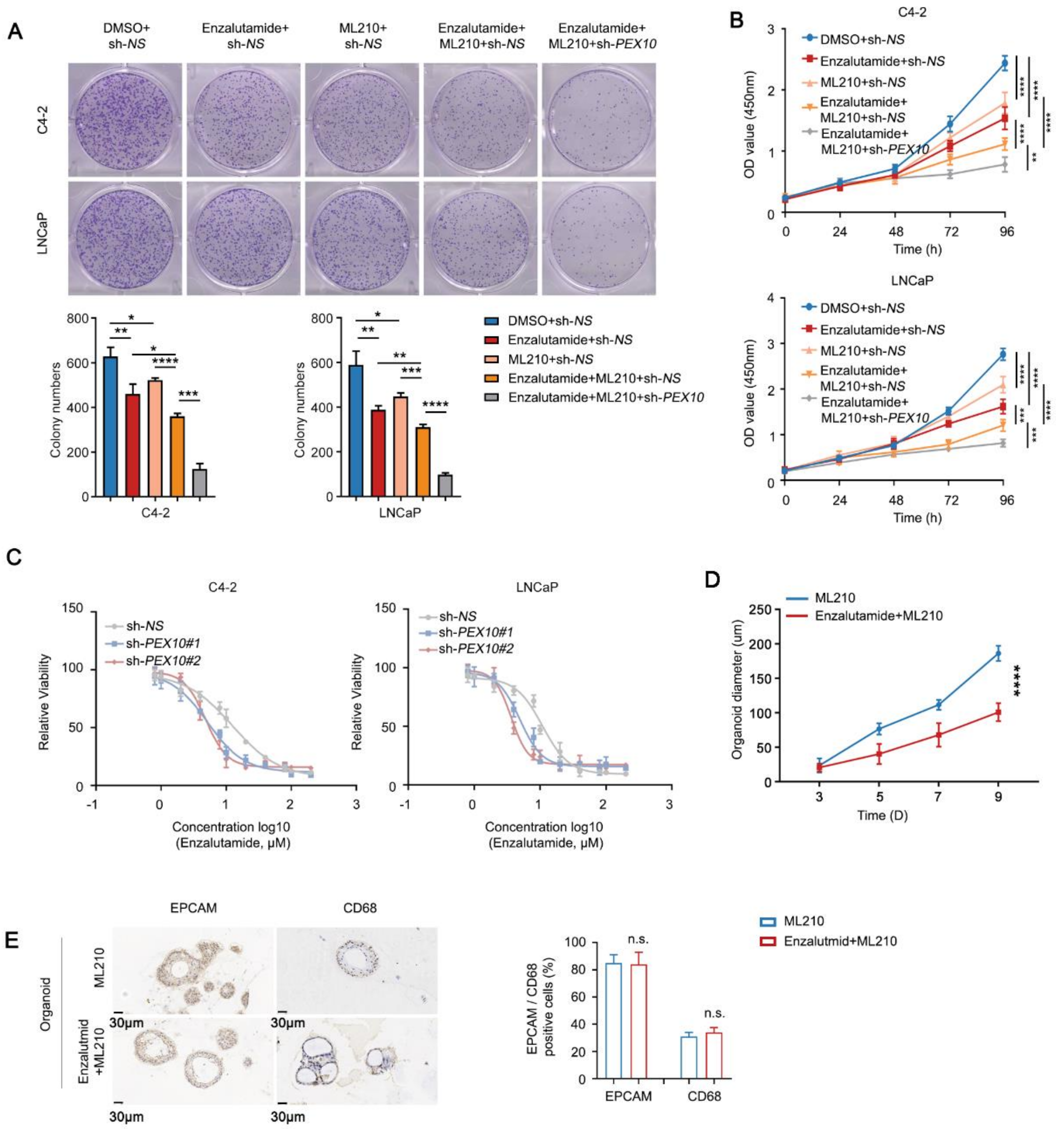

Supplementary Figure 7, Supplemental to Figure 6

A, Colony numbers of two *PEX10* knockdown or control prostate cancer cell lines (C4-2 and LNCaP) after treatment with ML210 (2μM) and combination with or without enzalutamide (5μM). Unpaired t-test. ($P < 0.05$ as "*"; $P < 0.01$ as "**"; $P < 0.001$ as "***"; $P < 0.0001$ as "****"). **B,** The CCK-8 OD value of two *PEX10* knockdown or control prostate cancer cell lines (C4-2 and LNCaP) after treatment with ML210 (2μM) and combination with or without enzalutamide (5μM). ANOVA. ($P < 0.01$ as "**"; $P < 0.001$ as "***"; $P < 0.0001$ as "****").

C, The IC50 in two prostate cell lines (C4-2 and LNCaP) after *PEX10* knockdown. **D,** Static of patient prostate cancer tissue original organoid after treatment with ML210 (2μM) or enzalutamide (5μM) combination. ANOVA. ($P < 0.0001$ as "****"). **E,** Expression and statistic of EPCAM and CD68 in organoids of different groups by IHC method. Unpaired t-test. (n.s. no specific).

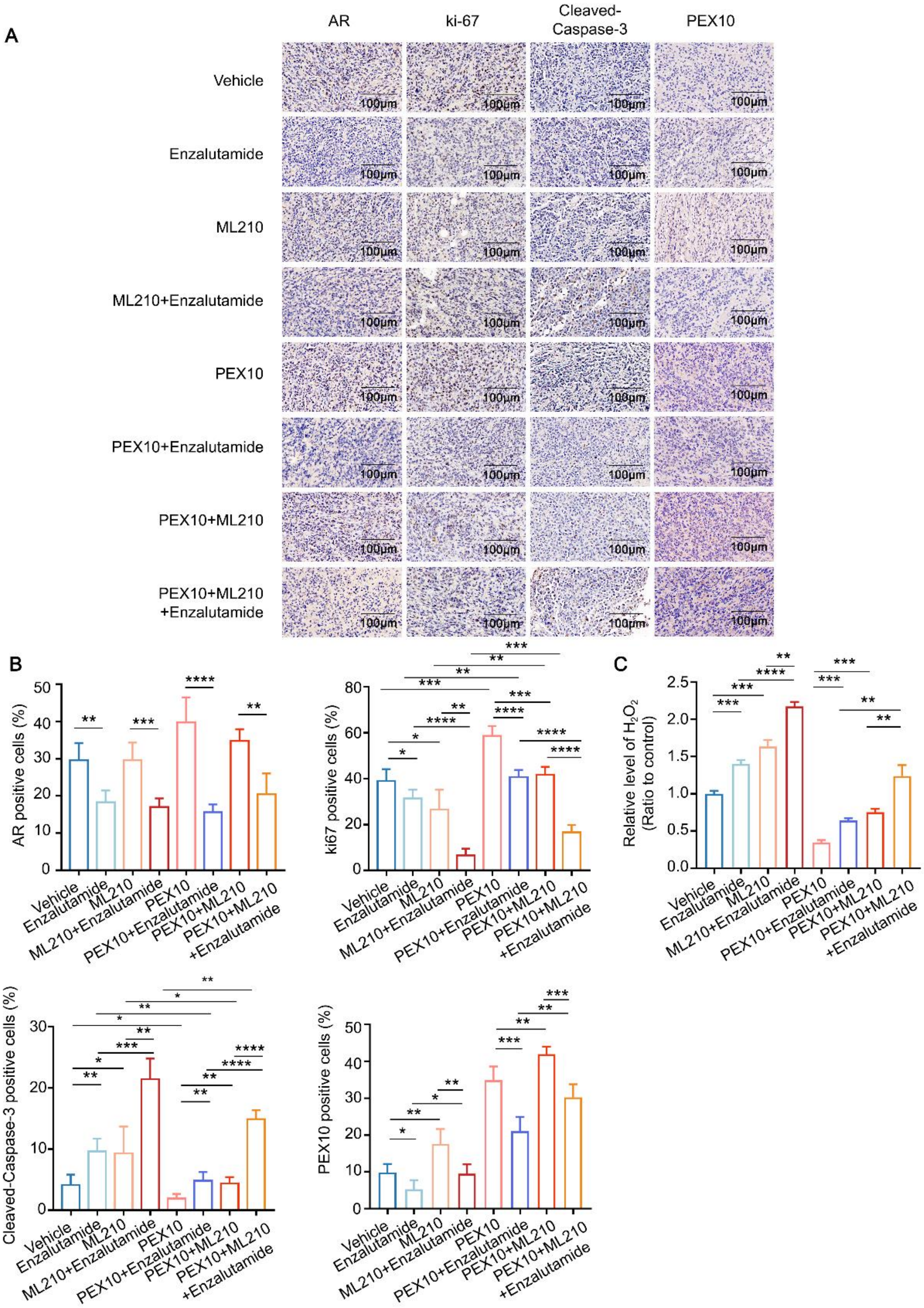

Supplementary Figure 8, Supplemental to Figure 6

A-B, Expression of AR, Ki67, Cleaved-Caspase-3 and PEX10 in xenografts of different groups by IHC method. All data expressed as mean ± S.E.M. Unpaired t-test. (P < 0.05 as

“*”; *P* < 0.01 as “**”; *P* < 0.001 as “***”; *P* < 0.0001 as “****”) **c,** H_2O_2 level in organoids of different groups. Unpaired t-test. (*P* < 0.05 as “*”; *P* < 0.01 as “**”; *P* < 0.001 as “***”).

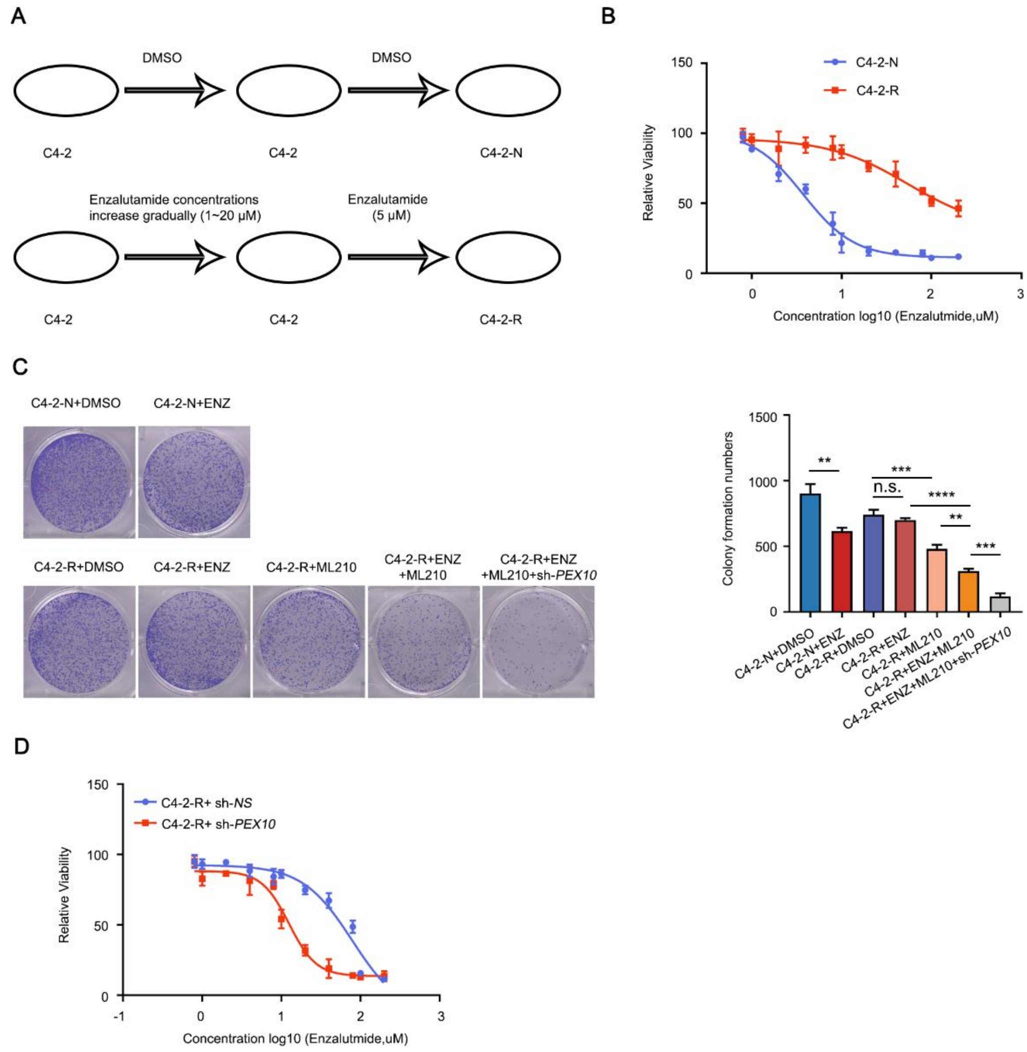

Supplementary Figure 9, Supplemental to Figure 6

A, Construction of the enzalutamide-resistant prostate cancer cell lines C4-2-R and the control group C4-2-N. **B,** The IC50 of enzalutamide in two prostate cell lines (C4-2-R and C4-2-N). **C,** Colony formation assay were performed on two cell lines (C4-2-R and C4-2-N) after treated with ML210 (2μM) alone or in combination with Enzalutamide (5μM) and (or) sh-*PEX10*. Unpaired t-test. (n.s. no specific; $P < 0.01$ as "**"; $P < 0.001$ as "***"; $P < 0.0001$ as "****"). **D,** The IC50 of enzalutamide in C4-2-R after knocked down *PEX10* or control.

Supplementary Table S1. Cell lines, antibodies and other reagents and resources

REAGENT or RESOURCE	SOURCE	IDENTIFIER
**Cell Lines**		
Human: C4-2	Bank of the Chinese Academy of Sciences	CRL-1435
Human: PC-3	Bank of the Chinese Academy of Sciences	HTB-81
Human: HEK293T	Bank of the Chinese Academy of Sciences	CRL-1740
Human: 22Rv1	Bank of the Chinese Academy of Sciences	CRL-2876
Human: LNCaP	Bank of the Chinese Academy of Sciences	CRL-1740
Human: VCaP	Bank of the Chinese Academy of Sciences	CRL-2876
Human: DU145	Bank of the Chinese Academy of Sciences	HTB-81
**Antibodies**		
Mouse monoclonal anti-beta Actin (WB: 1 μg/ml)	Abcam	Cat# ab8226
Mouse monoclonal anti-c-Myc (WB: 1/200)	Santa Cruz	Cat# sc-40
Mouse monoclonal anti-FLAG M2 (WB: 1/1000)	Sigma-Aldrich	Cat# F-3165
Mouse monoclonal anti-HA.11 (WB: 1/1000)	Covance	Cat# MMS-101R
Mouse monoclonal anti-His Probe (WB: 1/500)	Santa Cruz	Cat# SC-8036
Rabbit monoclonal anti-TFR (WB: 1/1000)	Abcam	Cat# ab214039
Rabbit polyclonal anti-TF (WB: 1/1000)	Thermofisher Scientific	Cat# PA3-913
Mouse monoclonal anti-FTH1 (WB: 1/200)	Santa Cruz	Cat# sc-376594
Rabbit polyclonal anti-PEX10 (WB: 1/1000; IHC: 1/100)	Thermofisher Scientific	Cat# PA5-116706
Rabbit monoclonal anti-AR (WB: 1/2000; IHC: 1/500)	Abcam	Cat# ab133273
Mouse monoclonal anti-FOXA1 (WB:1/1000)	Abcam	Cat# ab55178
Mouse polyclonal anti-AGPS (WB:1/1000)	Santa Cruz	Cat# sc-374201
Dylight 800, Goat Anti-Rabbit IgG (WB: 1/5000)	Abbkine	Cat# A23920
Dylight 800, Goat Anti-Mouse IgG (WB: 1/10000)	Abbkine	Cat# A23910

Rabbit polyclonal anti-PMP70 (IF: 5 µg/ml)	Abcam	Cat# ab3421
Rabbit monoclonal anti-AR (ChIP-qPCR)	Abcam	Cat# ab108341
Rabbit monoclonal anti-FOXA1 (ChIP-qPCR)	Abcam	Cat# ab170933
Rabbit monoclonal anti-AR (IHC: 100µl)	Fuzhou Maixin Biotech. Co., Ltd.	Cat# RMA-0807
Mouse monoclonal anti-CK5/6 (IHC: 100µl)	Fuzhou Maixin Biotech. Co., Ltd.	Cat# MAB-0744
Mouse monoclonal anti-ki-67 (IHC: 100µl)	Fuzhou Maixin Biotech. Co., Ltd.	Cat# MAB-0672
Rabbit polyclonal anti-PEX5 (WB:1/1000)	Solarbio	Cat# K004208P
Rabbit polyclonal anti-MBOAT2 (WB:1/1000)	Novus Biologicals	Cat# NBP182236
Mouse monoclonal anti-GPX4 (WB:1/200)	Santa Cruz	Cat# sc-166570;
Mouse monoclonal anti-MLKL (WB:1/200)	Santa Cruz	Cat# sc-293201
Rabbit monoclonal anti-p-MLKL (WB:1/1000)	Abcam	Cat# ab187091
Mouse monoclonal anti-CD68 (IHC:1/50)	Santa Cruz	Cat# sc-70761
Mouse monoclonal anti- EPCAM (IHC:1/50)	Santa Cruz	Cat# sc-59783
**Bacterial and Virus Strains**		
E. coli DH5α	Thermo Fisher	Cat#18258012
E. coli BL21	Thermo Fisher	Cat# C600003
**Chemicals, Peptides, and Recombinant Proteins**		
ML210	MedChemExpress	Cat# HY-100003
RSL3	Solarbio	Cat# IR1120
Enzalutamide	Solarbio	Cat# IE0760
FAC	Solarbio	Cat# A9181
Lipofectamine 2000 reagent	Thermo Fisher	Cat# 11668500
NAC	MedChemExpress	Cat# HY-B0215
BIBR 1532	MedChemExpress	Cat# HY-17353
B27	Thermo Fisher	Cat# 17504044
A83-01	MedChemExpress	Cat# HY-10432
Y-27632	MedChemExpress	Cat# HY-10071

Activin A	Novoprotein	Cat# No.:C687
RSPO1	Novoprotein	Cat# No.:CX83
EGF	Novoprotein	Cat# No.:C029
FGF-10	Novoprotein	Cat# No.:CR11
Noggin	Novoprotein	Cat# No.:CB89
FGFb	Novoprotein	Cat# No.:C779

Supplementary Table S2. Sequence information of shRNAs

**Gene**	**Sequence (5’-3’)**
Sh-control	5′- -3′ CAACAAGATGAAGAGCACCAA
Sh-*PEX10* -#1	5′- -3′ GTTGAGCTGCTCTCAGATGTT
Sh-*PEX10* -#2	5′- -3′ GAGTACGTCAGCATCATCCAT
Sh-*AR*-#1	5′- -3′ CTGAAGAAACTTGGTAATCTT
Sh-*AR*-#2	5′- -3′ CGTGGACTTTCCGGAAATGAT
Sh-*FOXA1*-#1	5′- -3′ CTCCATGAACTCCATGAACAT
Sh-*FOXA1*-#2	5′- -3′ CACTGCAATACTCGCCTTACT

Supplementary Table S3. Sequence information of primers for RT-qPCR

**Species**	**Gene**	**Forward (5’-3’)**	**Reverse (5’-3’)**
Human	*GAPDH*	TGACATCAAGAAGGTGGTGAAGCAG	GTGTCGCTGTTGAAGTCAGAGGAG
Human	*KLK3*	TCCCACACCCGCTCTACGATATG	GTCCATGACCTTCACAGCATCCG
Human	*PEX3*	TCTTCCTGGGCACGGTCCTTG	GCAGCCTCCCTTTCCTGTATTTCTC
Human	*PEX5*	ACCCAGGCAGAGAATGAACAAGAAC	GCAGGGACTCGTTGGTGAAGC
Human	*PEX7*	GGATGTGAAGGCAGCAGGAGTAAG	CCCAGCCTCTCAAACTACAGTCAAC
Human	*PEX10*	AAGTGGCTGGAGTGGAGGAAGG	CACCTGGATGATGCTGACGTACTC
Human	*PEX12*	GATGCAGCAACCAGCCAGGAG	CCAGTAGACAGGGATAAGGCAACAC
Human	*PEX13*	GCCATCACAGCAGACAGGAAGTAG	CGGTTGTAGCCCAGCCCATTATATC

Human	*AGPS*	TGGAGGGAGCCTGTCACATCAC	GCATCCCAAAGCCGACATCAGAG
Human	*FAR1*	GTTGCTGAGGTCTTGTCCTAAGGTG	CACTCGCTCTTGTGGTGTCTGTC
Human	*AR*	CTACATCAAGGAACTCGATCGT	CATGTGTGACTTGATTAGCAGG
Human	*FOXA1*	CTACTACGCAGACACGCAGG	CCGCTCGTAGTCATGGTGTT
