# Supplementary material for: Enzalutamide inhibits PEX10 function and sensitizes prostate cancer cells to ROS activators
Source: Cell Death Dis. 2024 Aug 3;15(8):559. doi: 10.1038/s41419-024-06937-7 (PMC11297951; doi:10.1038/s41419-024-06937-7)
Supplement: Supplementary file 2 — Original western blots [file 41419_2024_6937_MOESM2_ESM.pdf]

Figure 3A

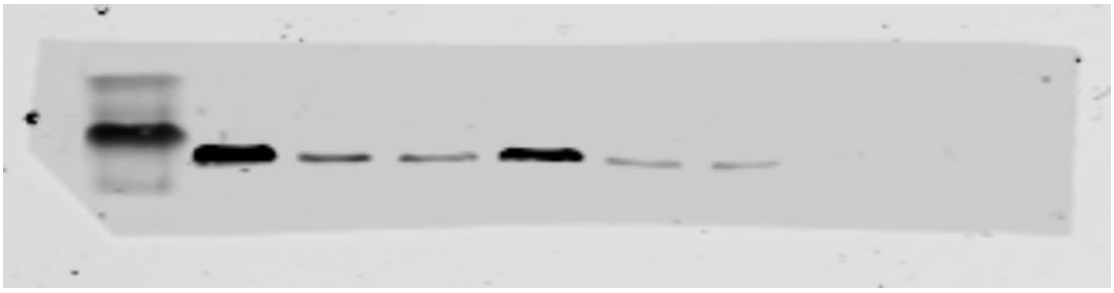

PEX10

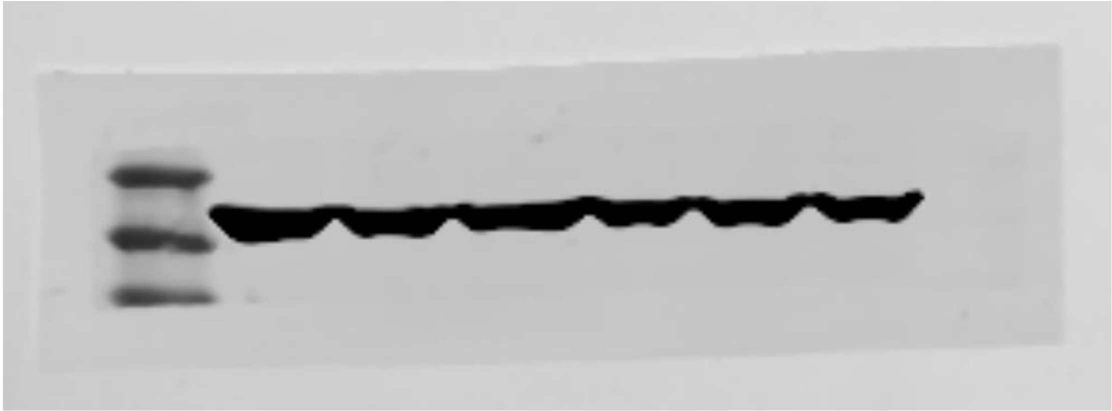

β-Actin

Figure 4B

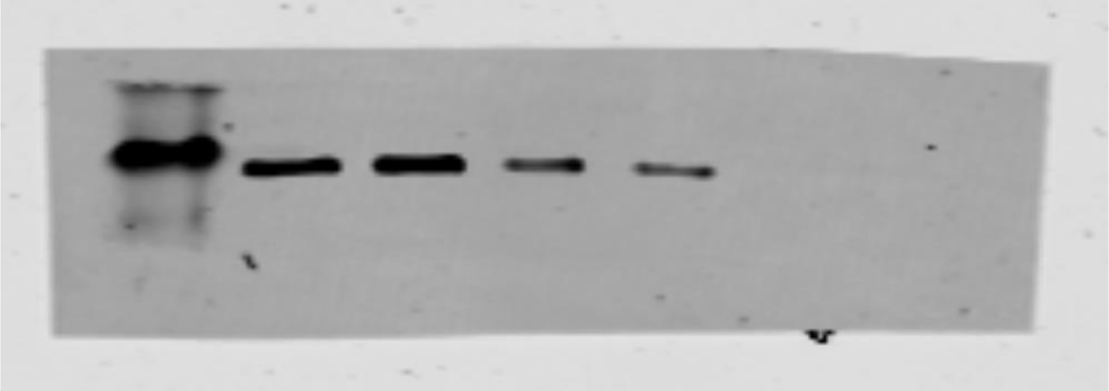

PEX10

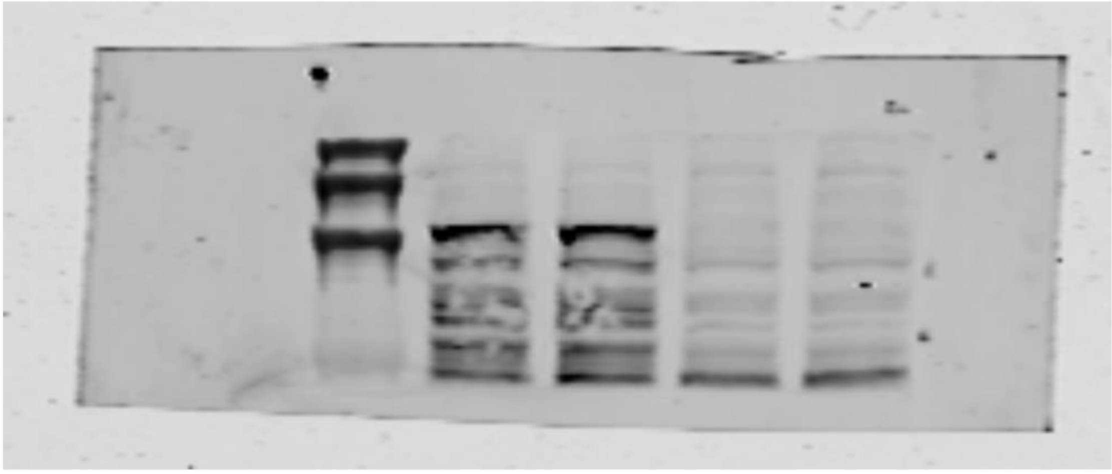

AR

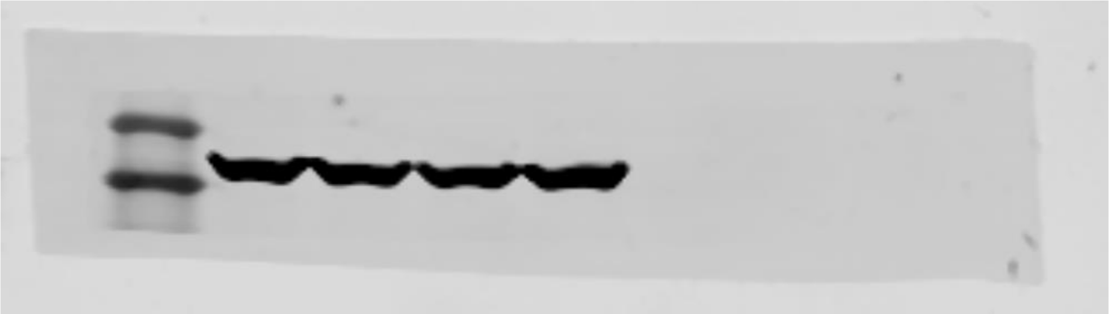

β-Actin

Figure 4E

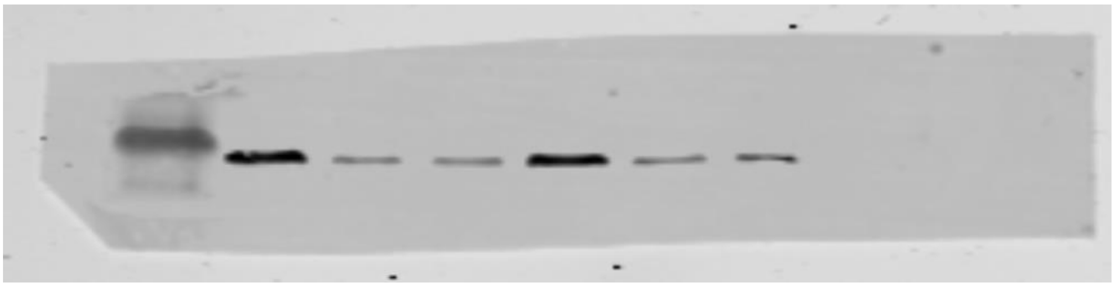

PEX10

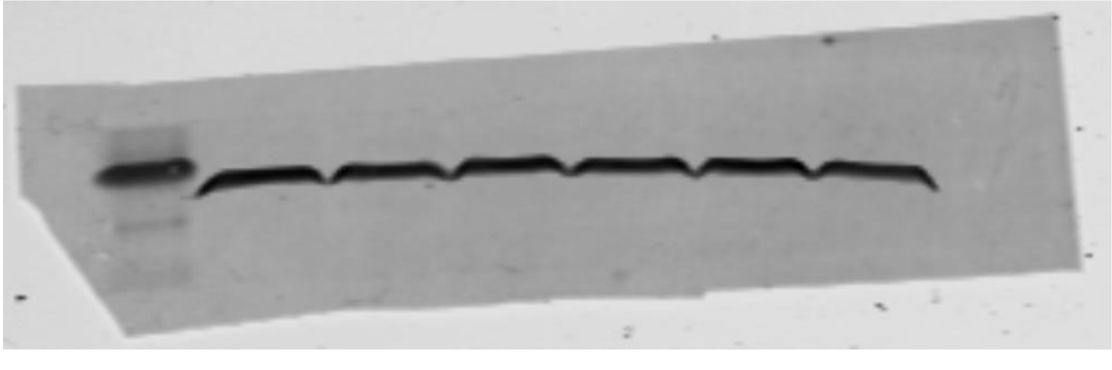

PEX5

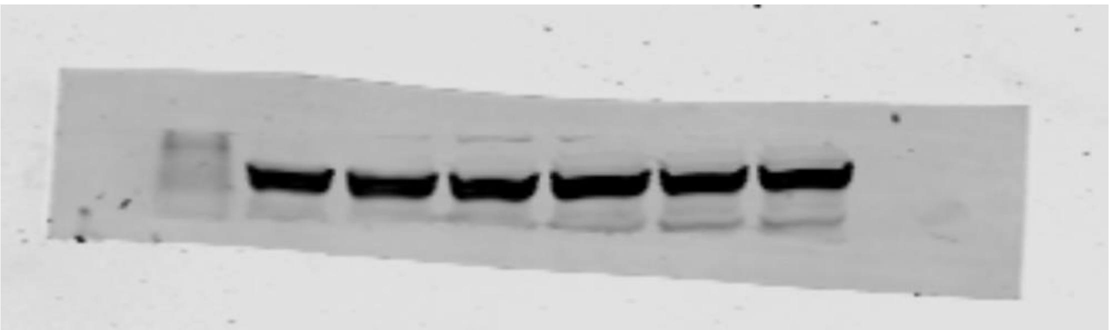

AGPS

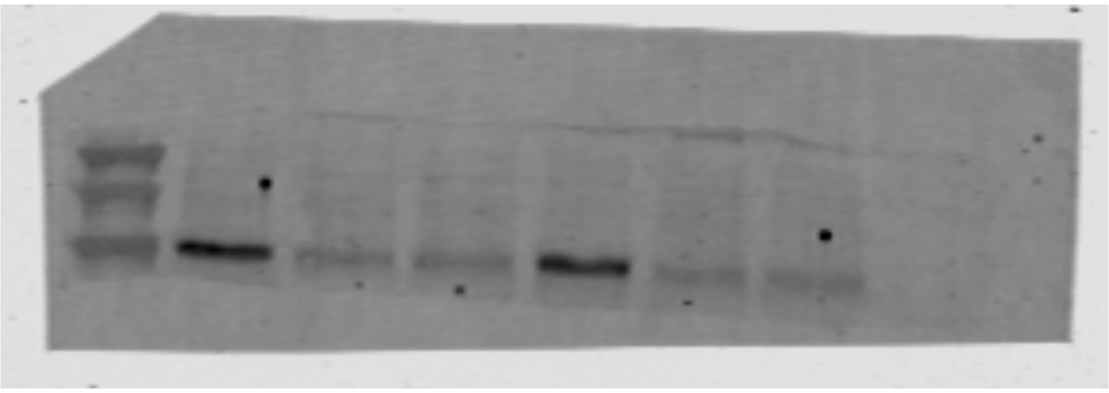

AR

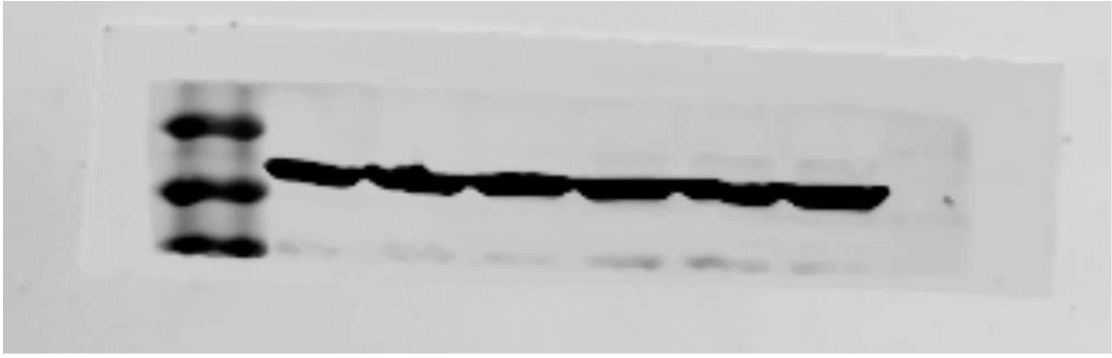

β-Actin

Figure 4N

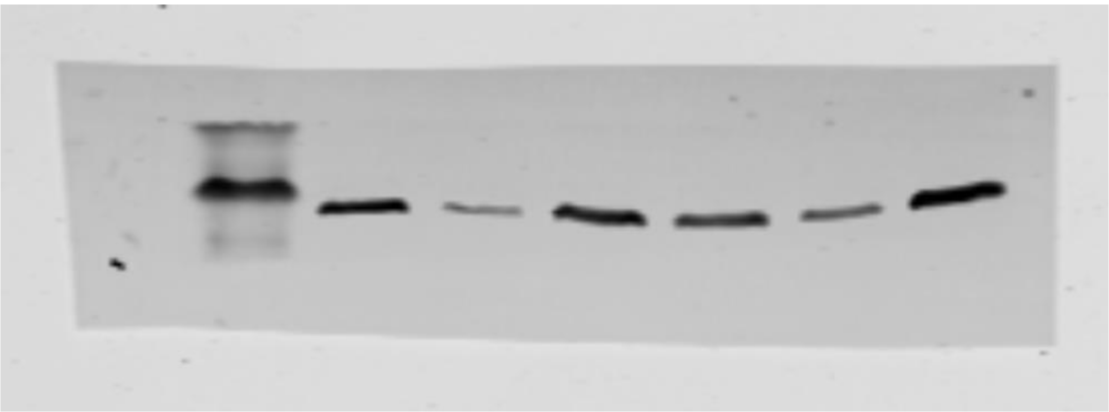

PEX10

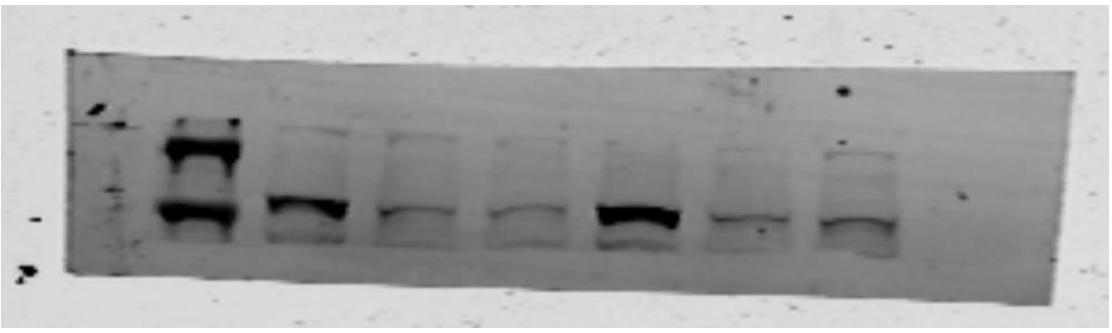

AR

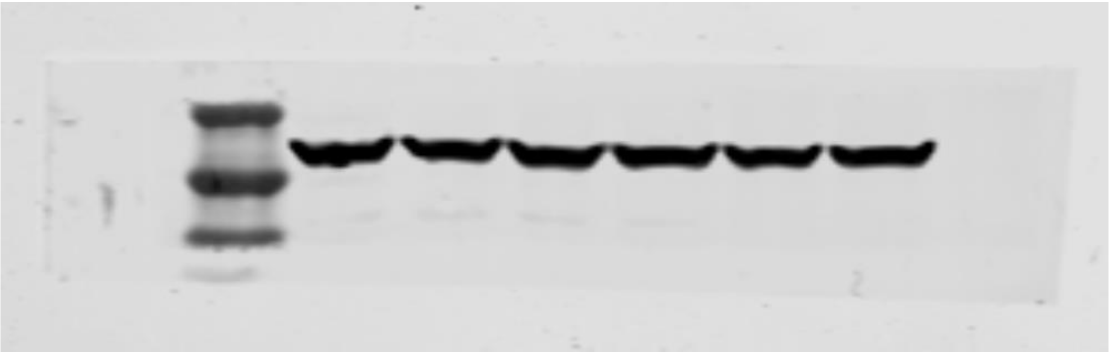

β-Actin

Figure 5C

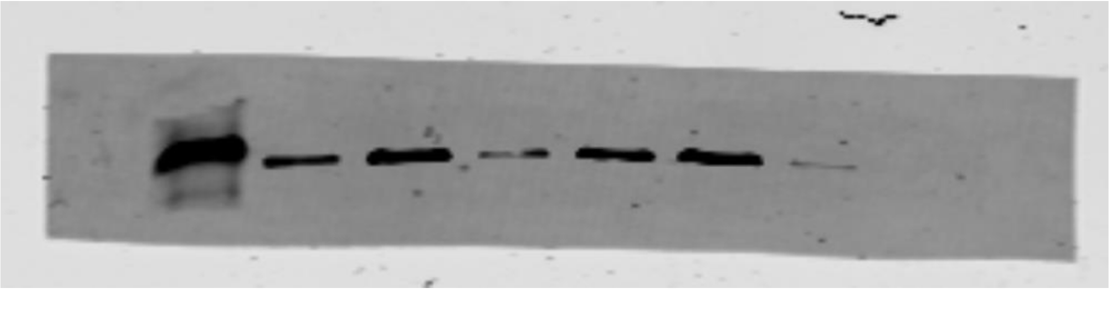

PEX10

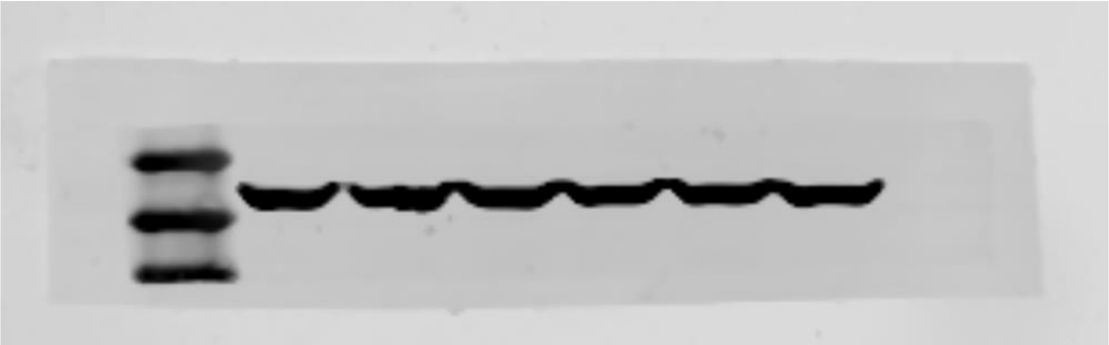

β-Actin

Figure 5J

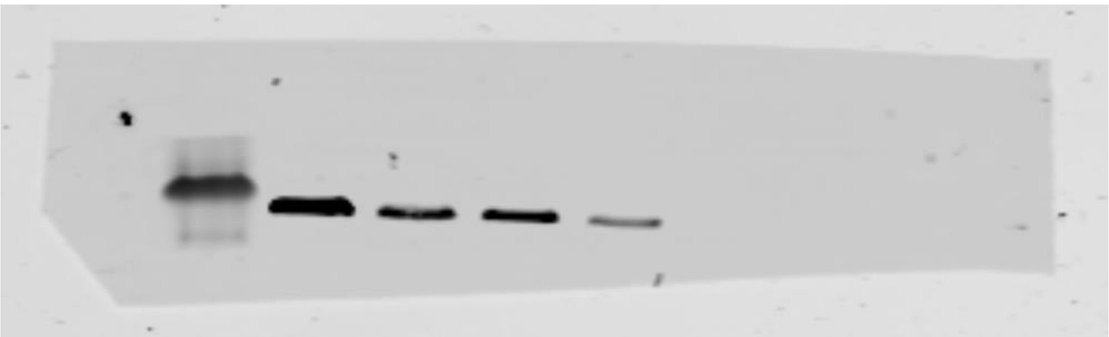

LNCaP PEX10

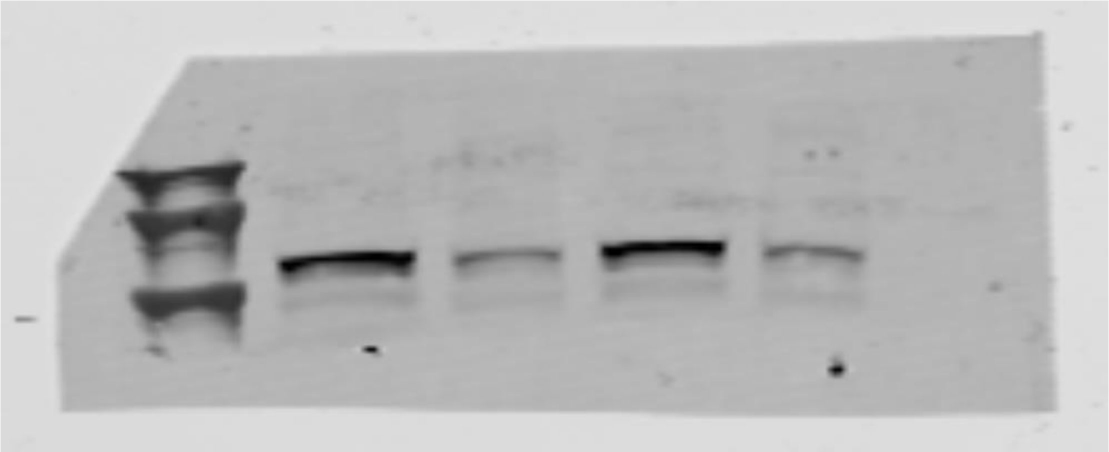

LNCaP AR

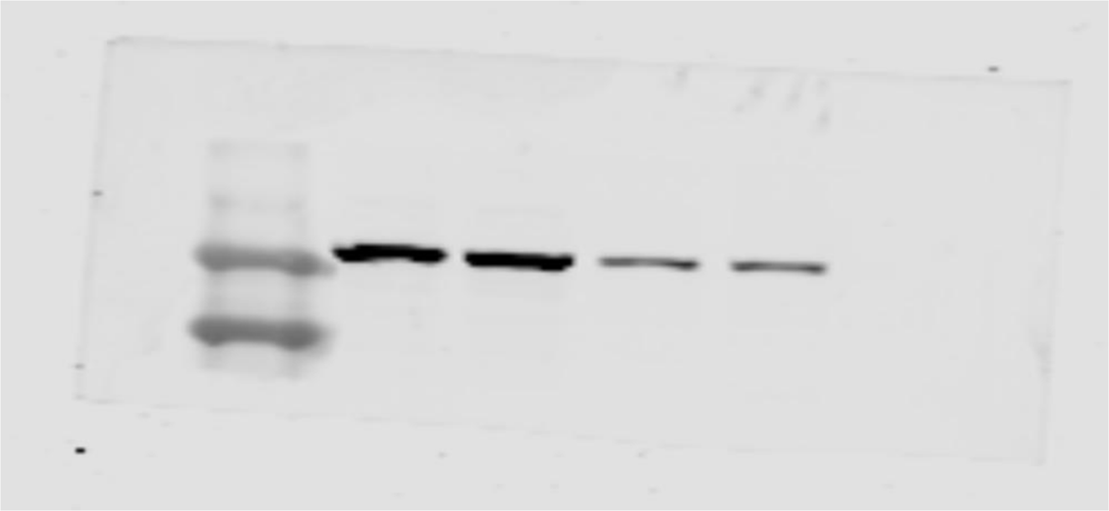

LNCaP FOXA1

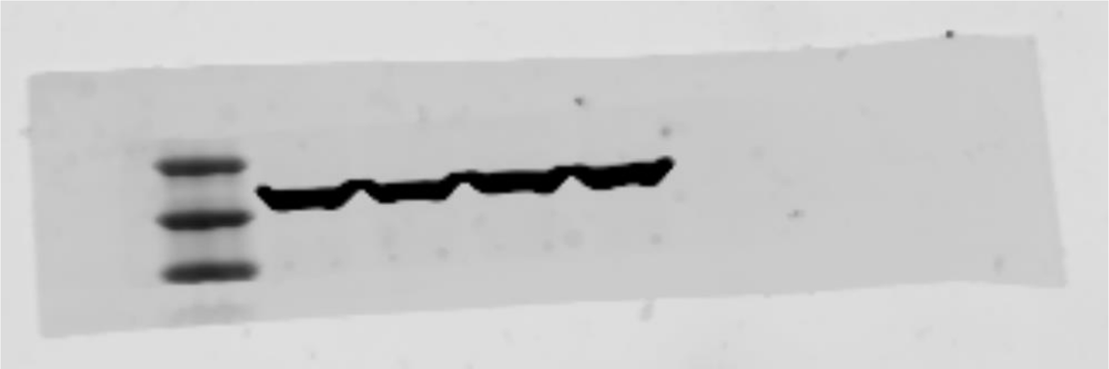

LNCaP β-Actin

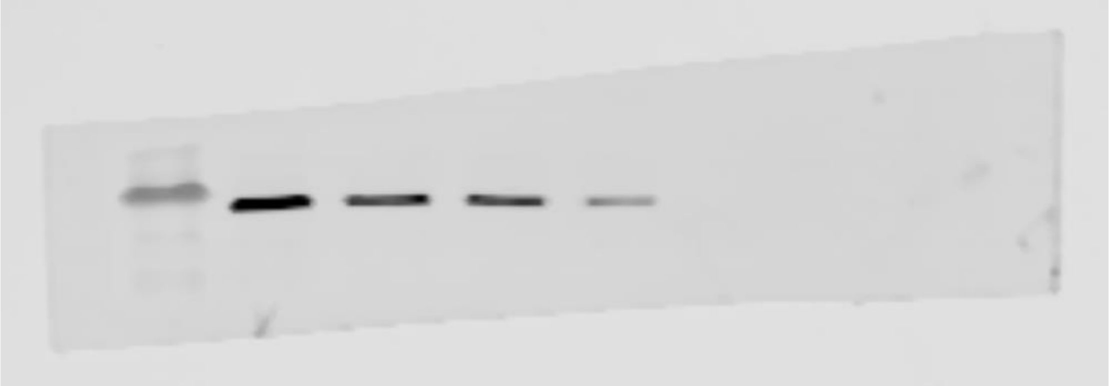

C4-2 PEX10

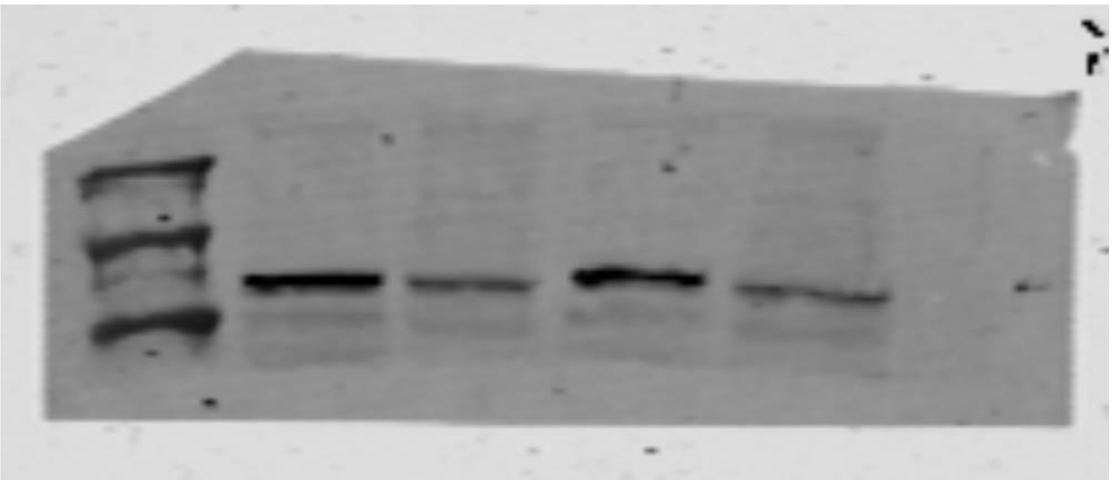

C4-2 AR

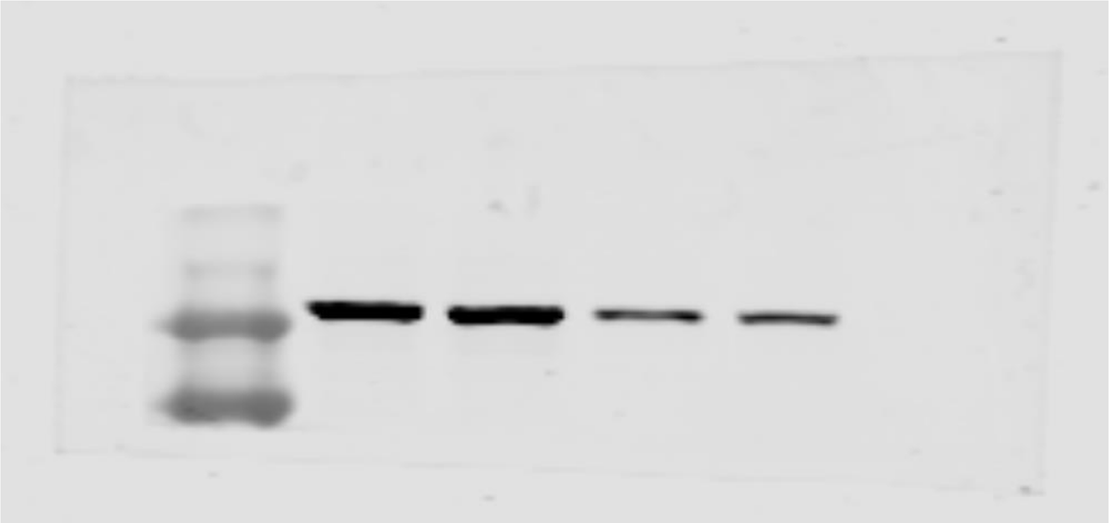

C4-2 FOXA1

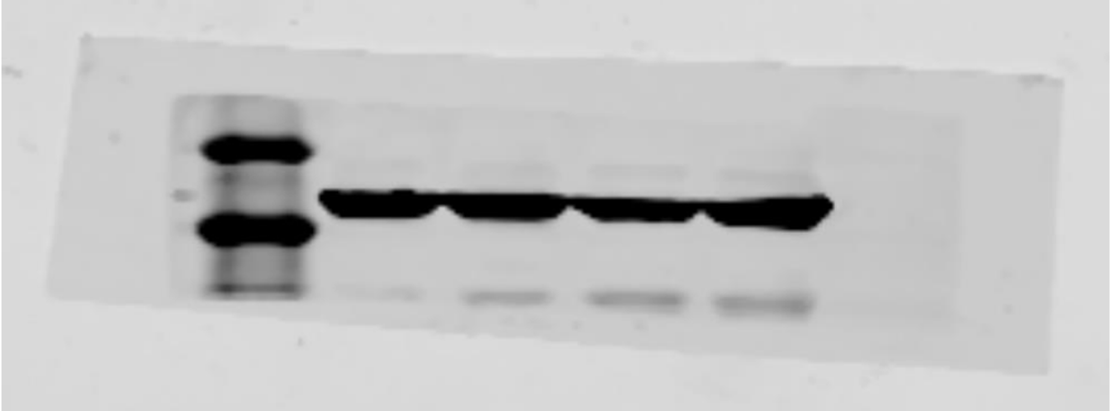

C4-2 β-Actin

Figure 5K

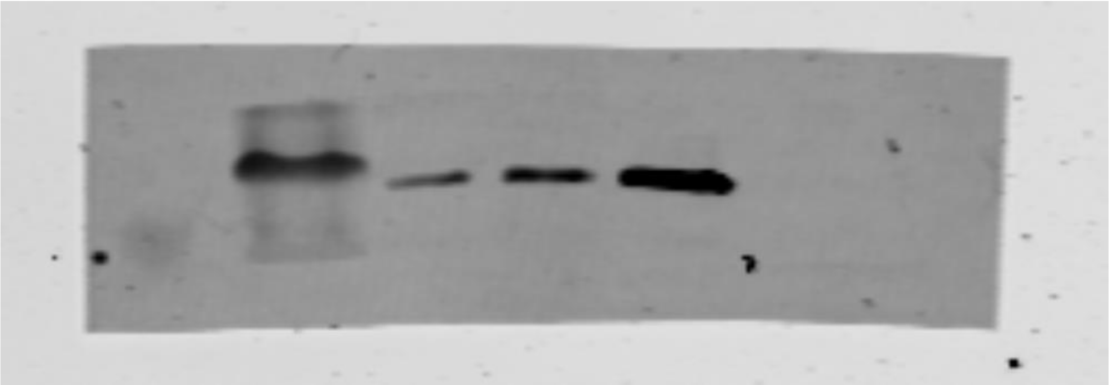

LNCaP PEX10

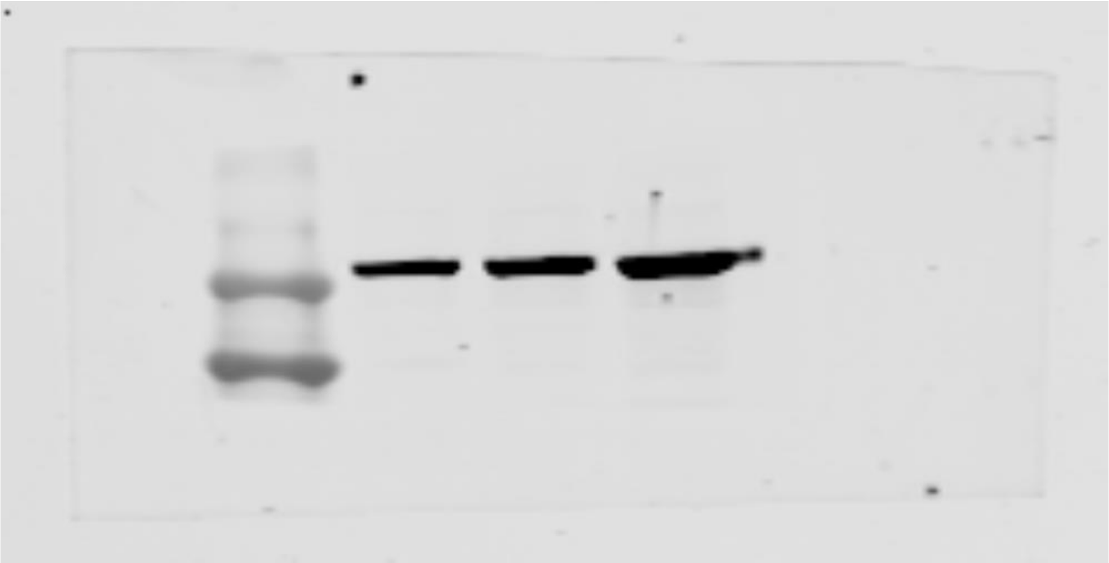

LNCaP FOXA1

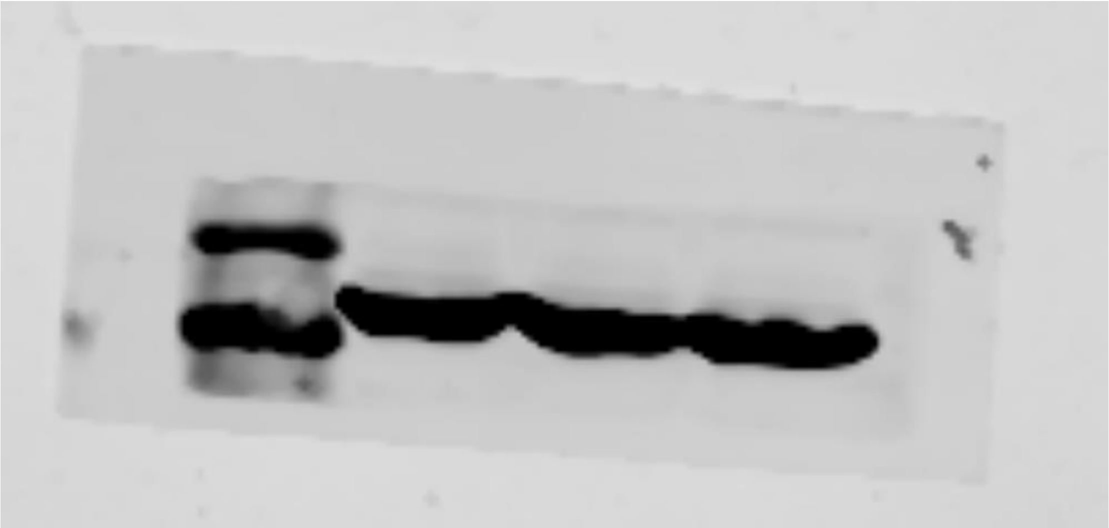

LNCaP β-Actin

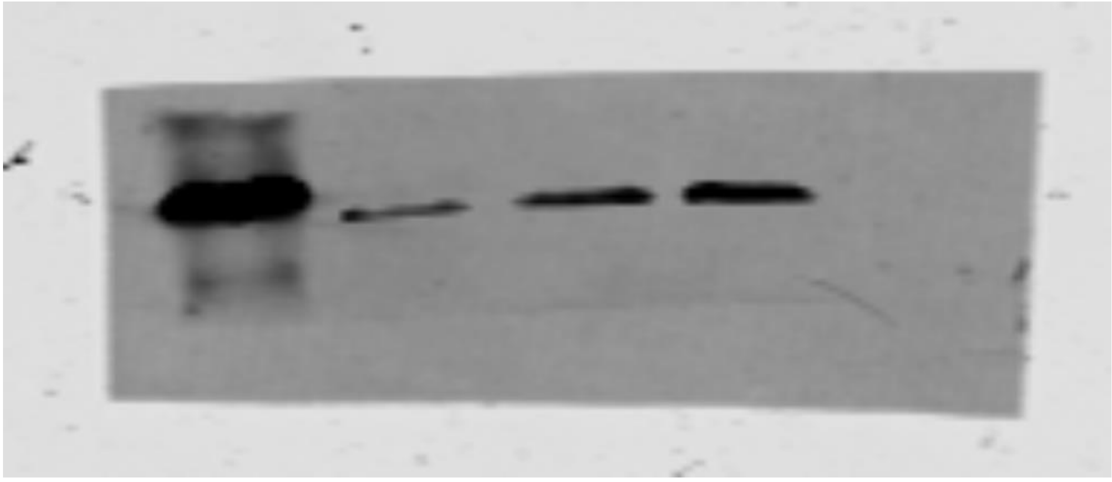

C4-2 PEX10

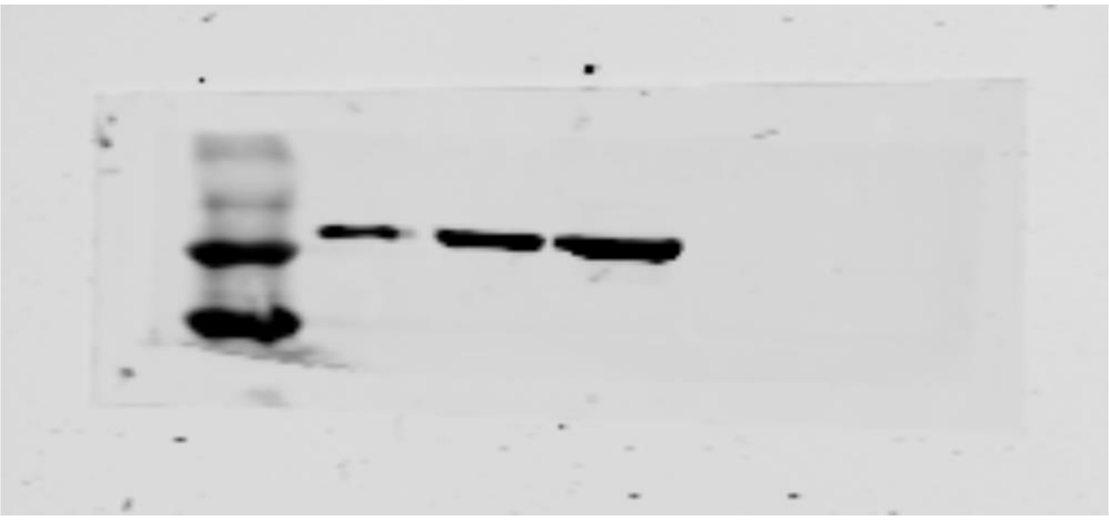

C4-2 FOXA1

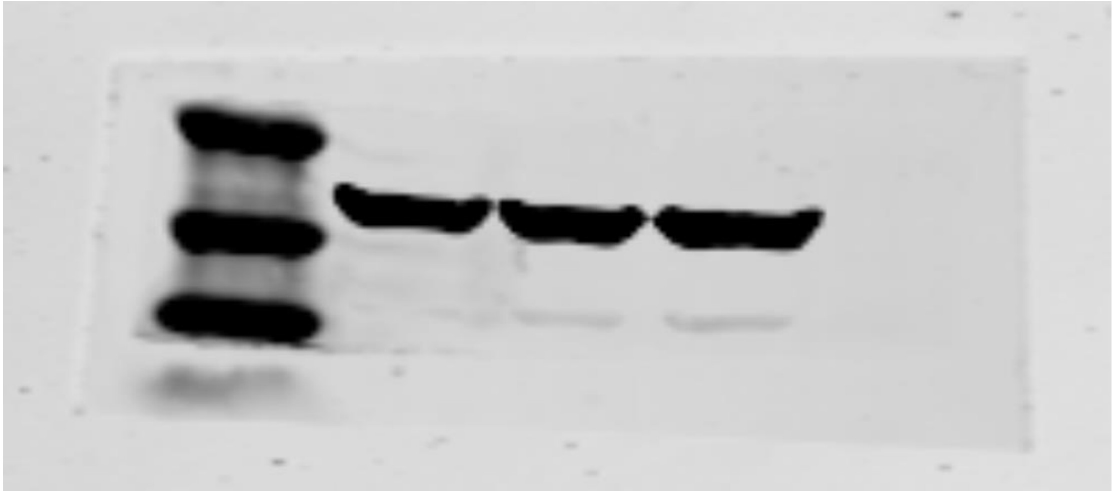

C4-2 β-Actin

Supplementary Figure 2A

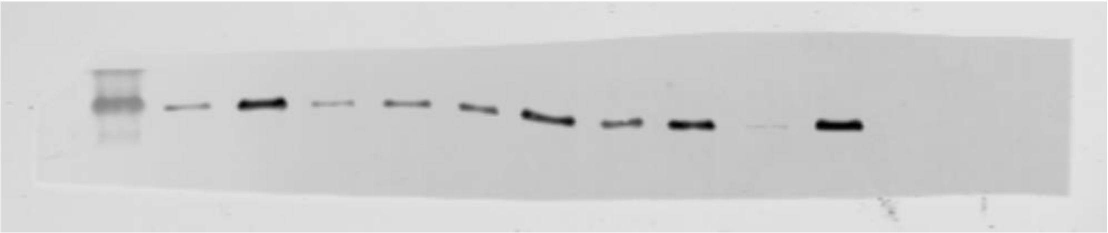

Patient 1-5   PEX10

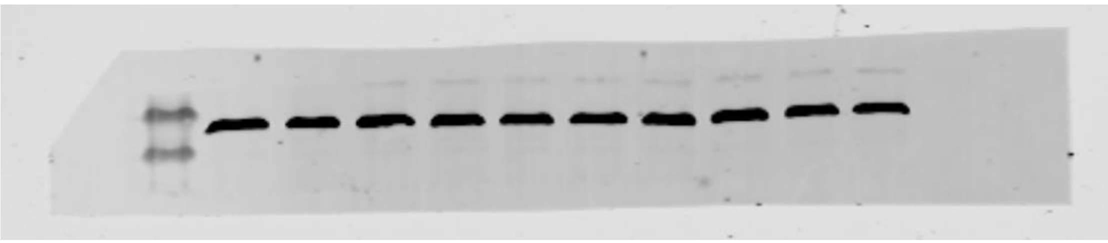

Patient 1-5   β-Actin

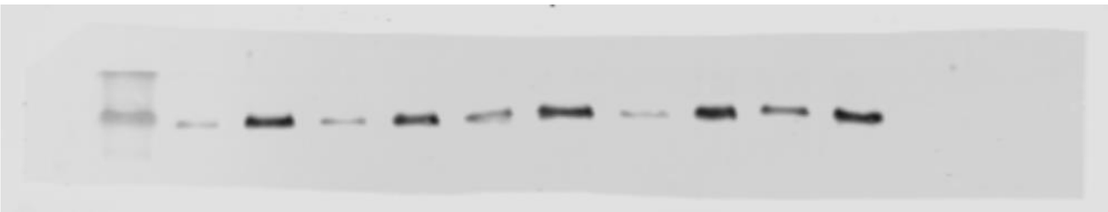

Patient 6-10   PEX10

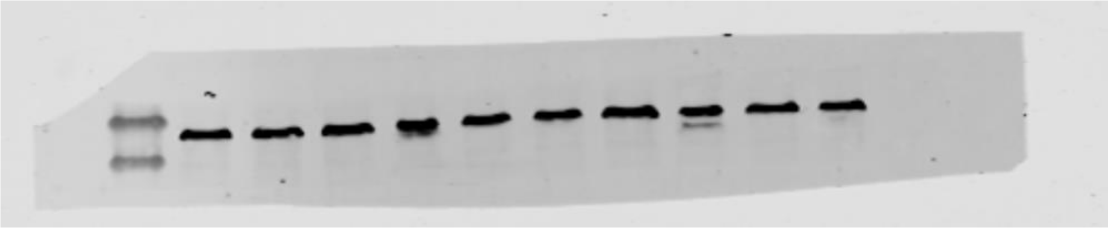

Patient 6-10   β-Actin

Supplementary Figure 4E

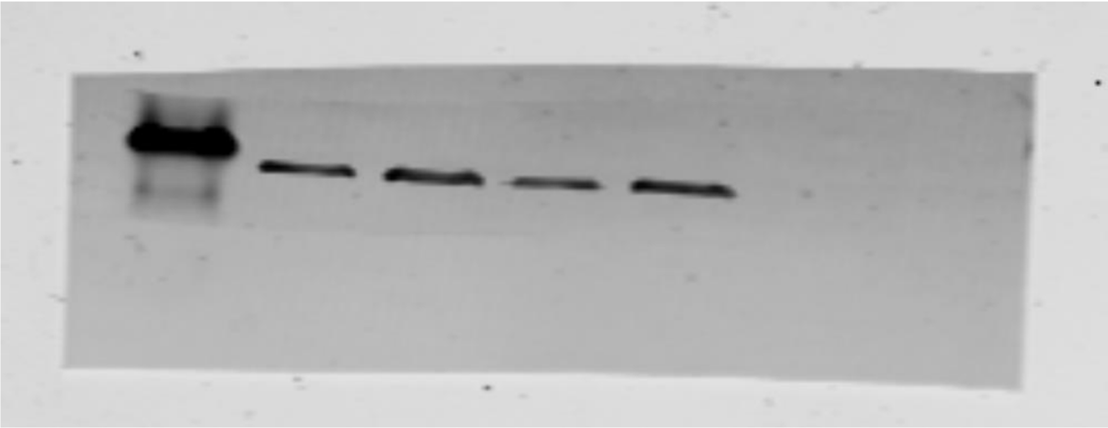

PEX10

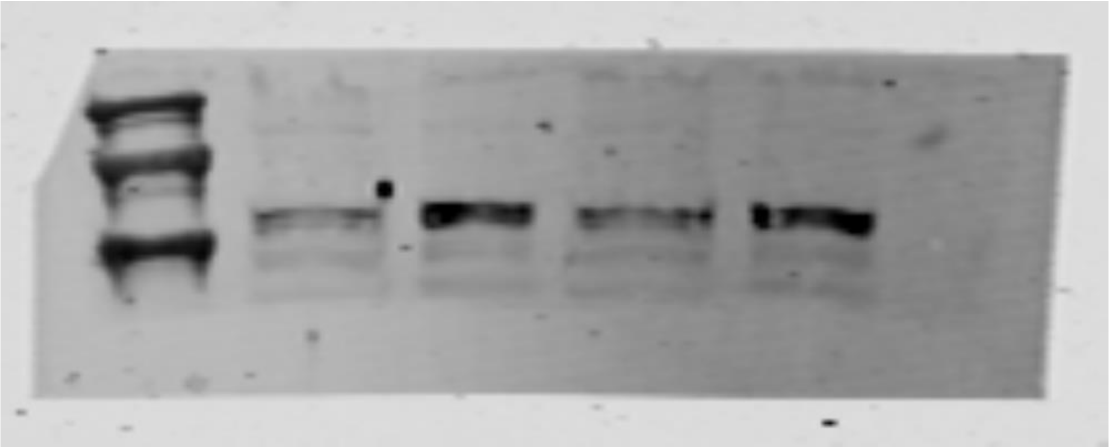

AR

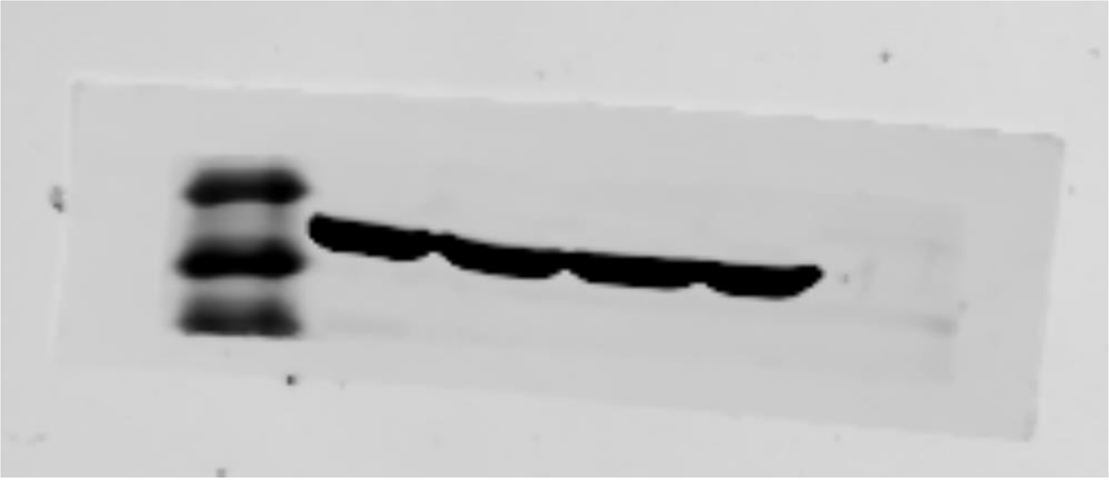

β-Actin

Supplementary Figure 5D

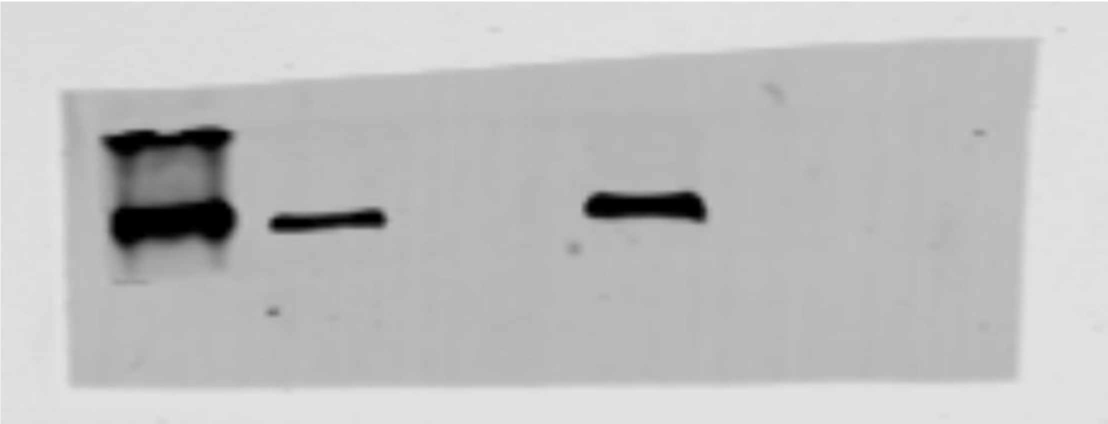

HA

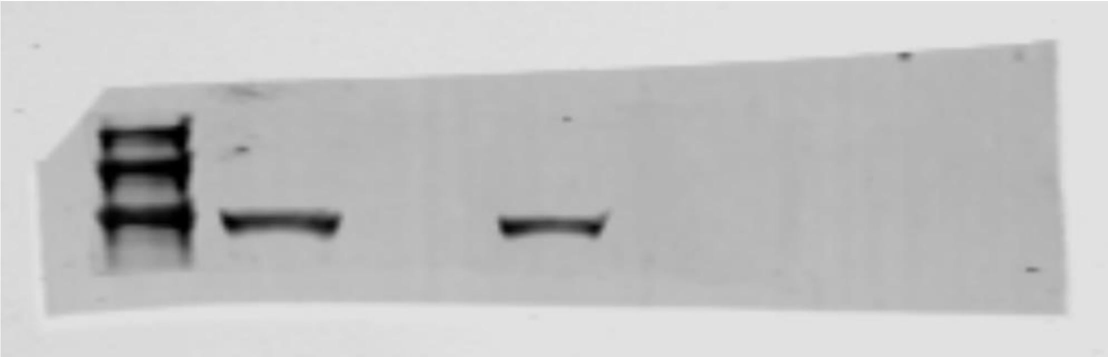

AR

Supplementary Figure 6B

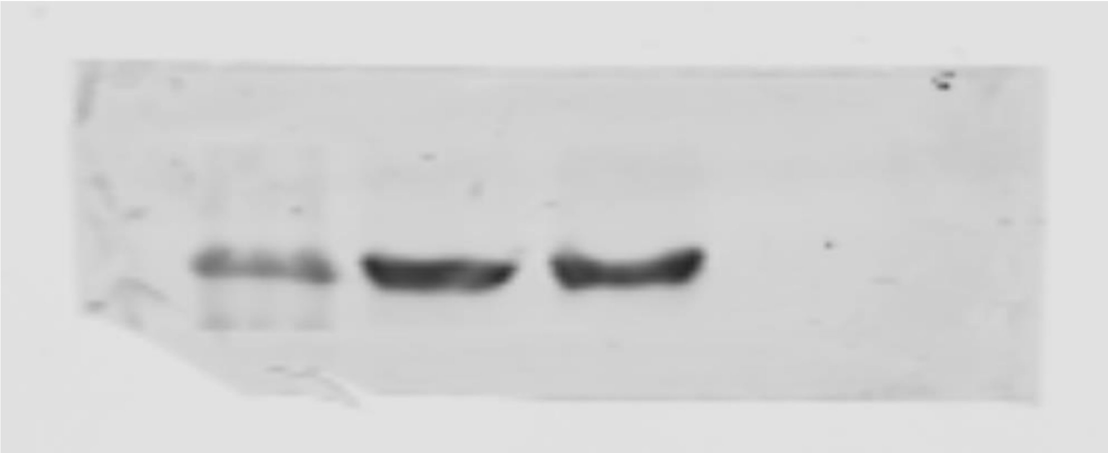

C4-2 MLKL

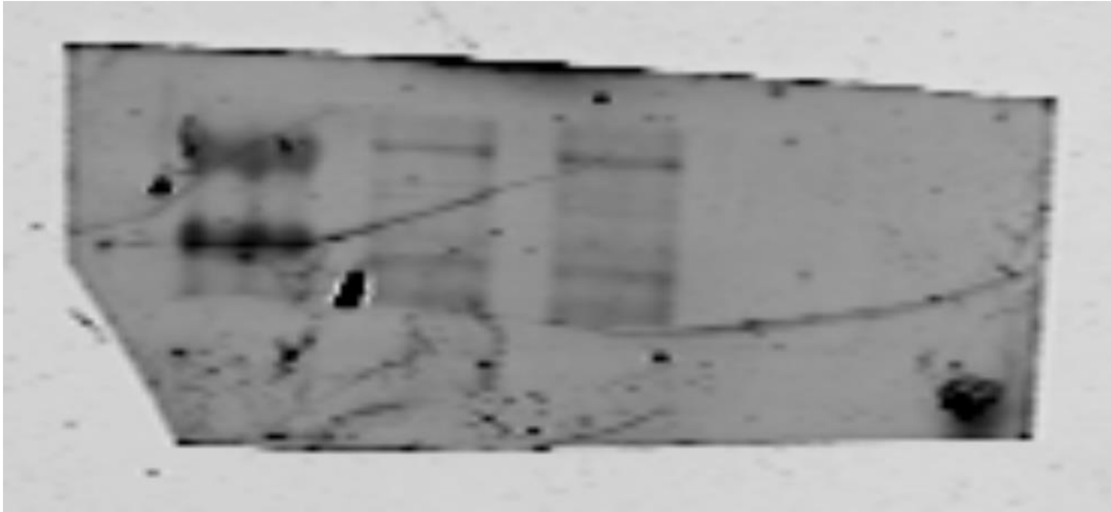

C4-2 p-MLKL

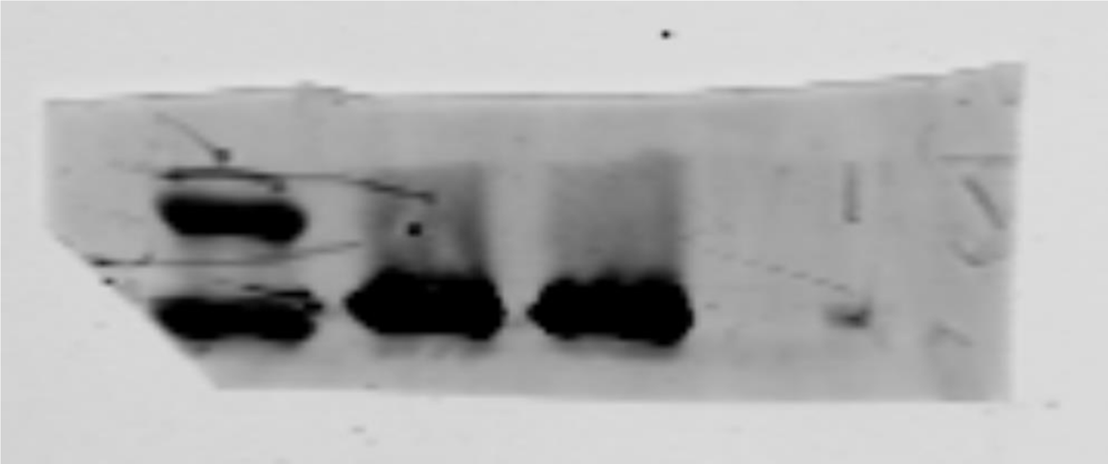

C4-2 β-Actin

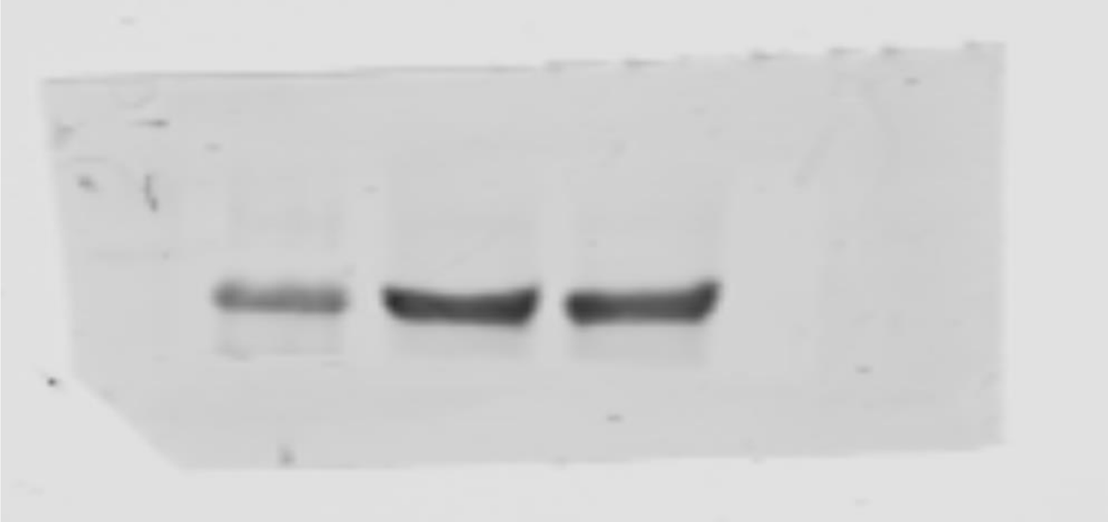

LNCaP MLKL

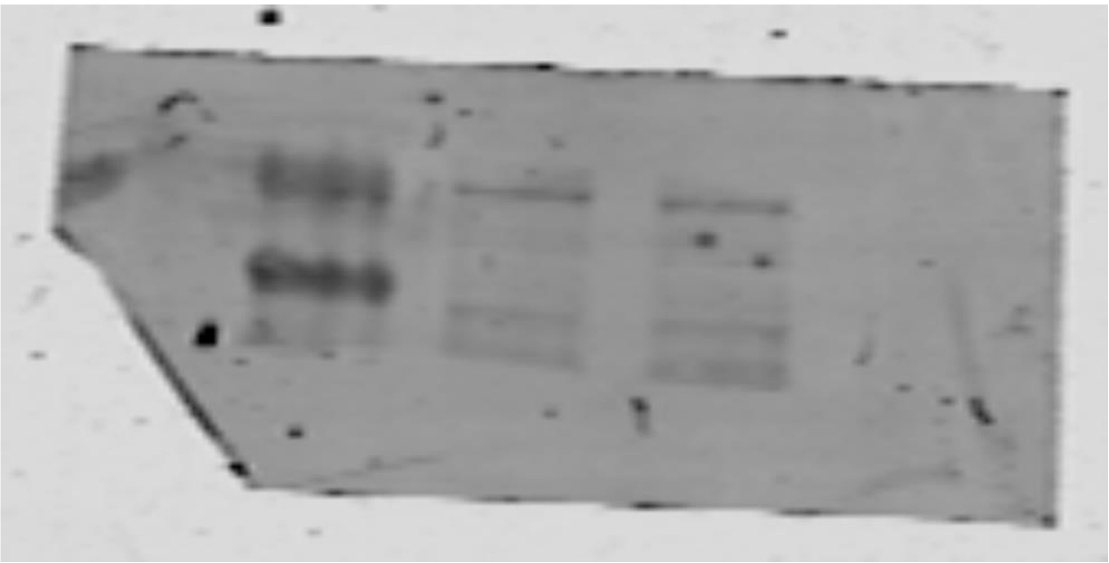

LNCaP p-MLKL

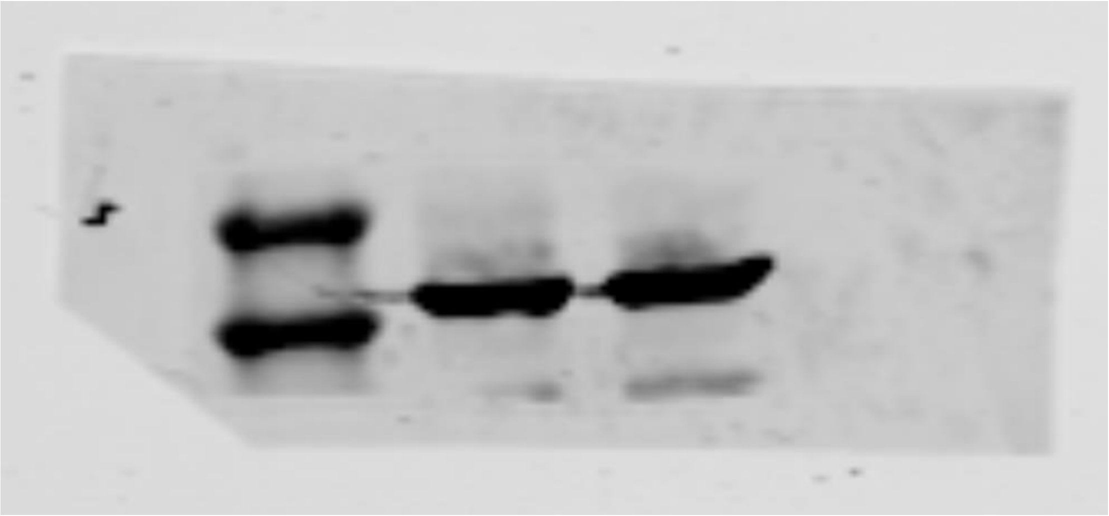

LNCaP β-Actin

Supplementary Figure 6C

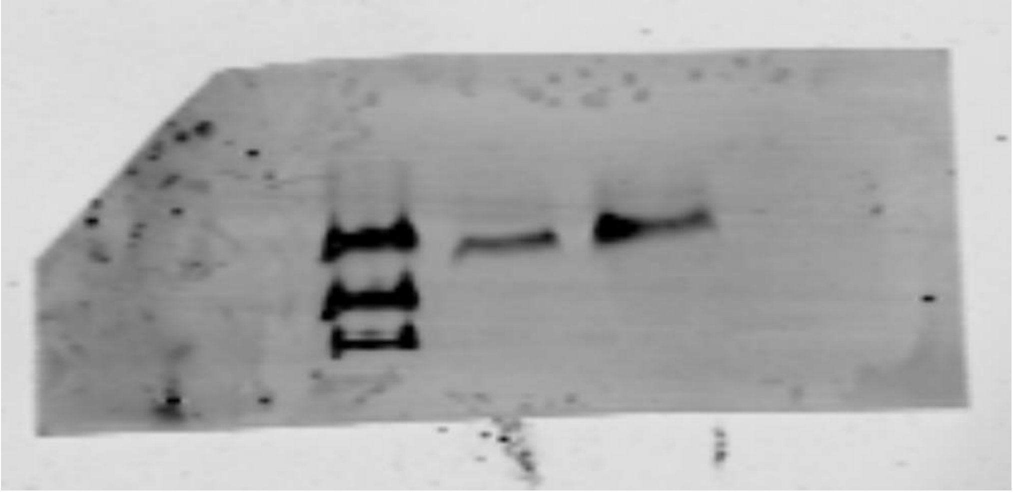

C4-2 AR

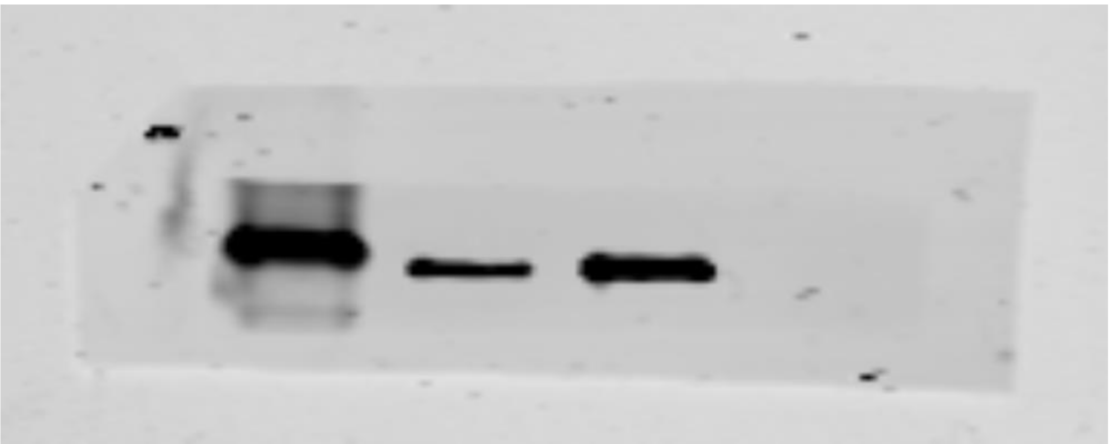

C4-2 PEX10

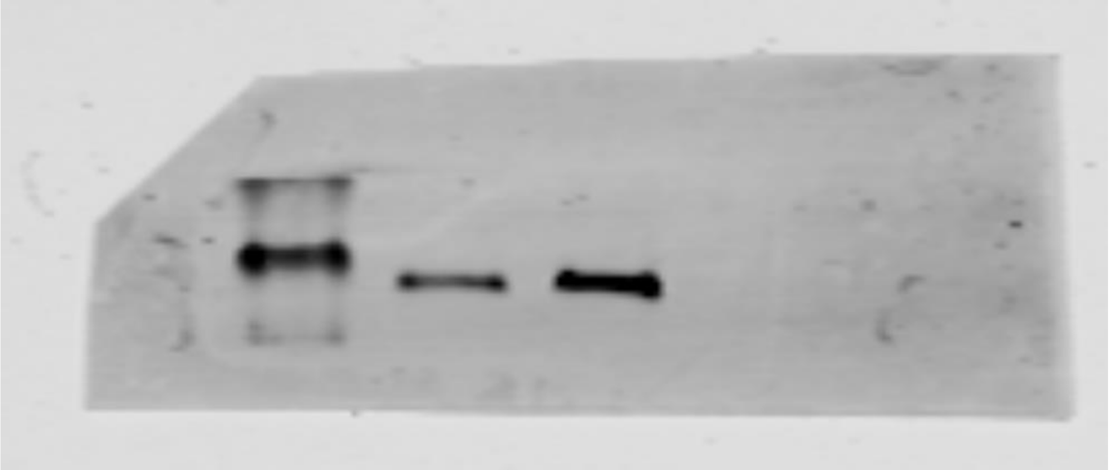

C4-2 MBOAT2

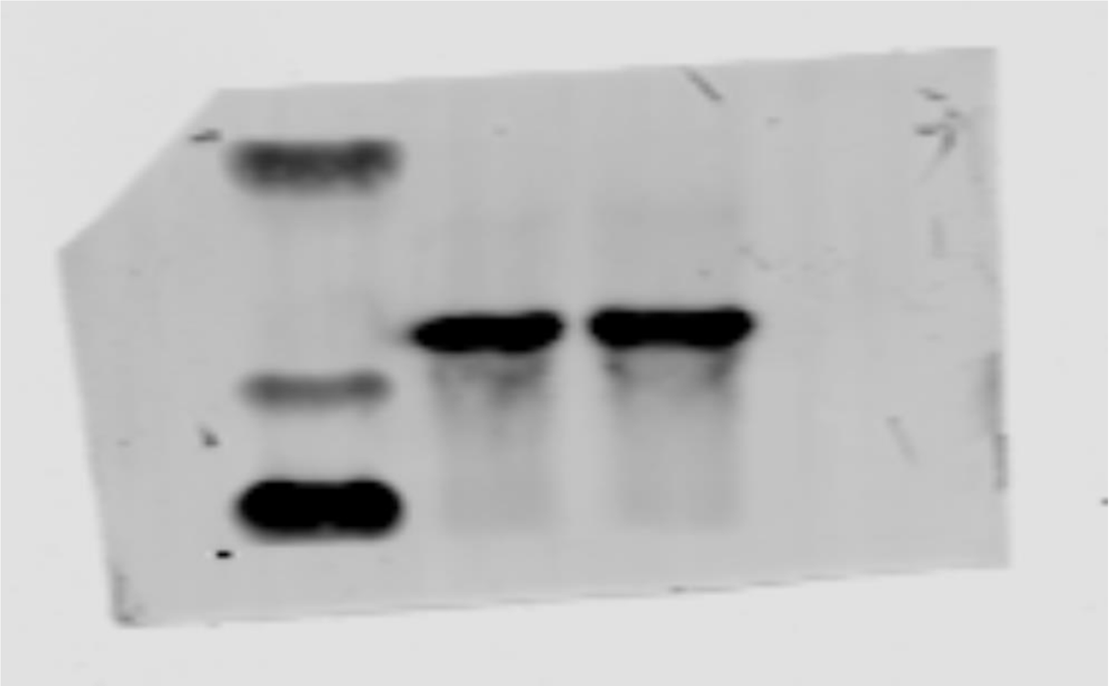

C4-2 GPX4

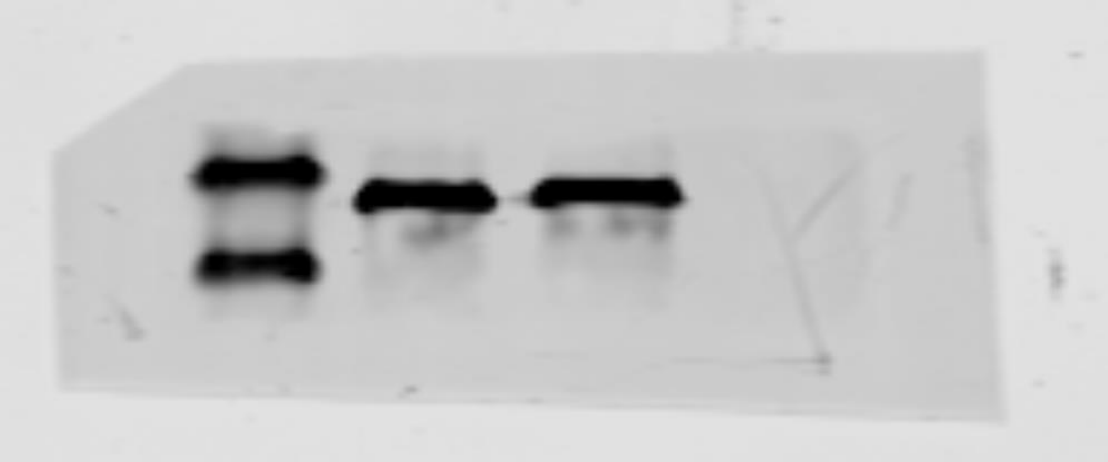

C4-2 β-Actin

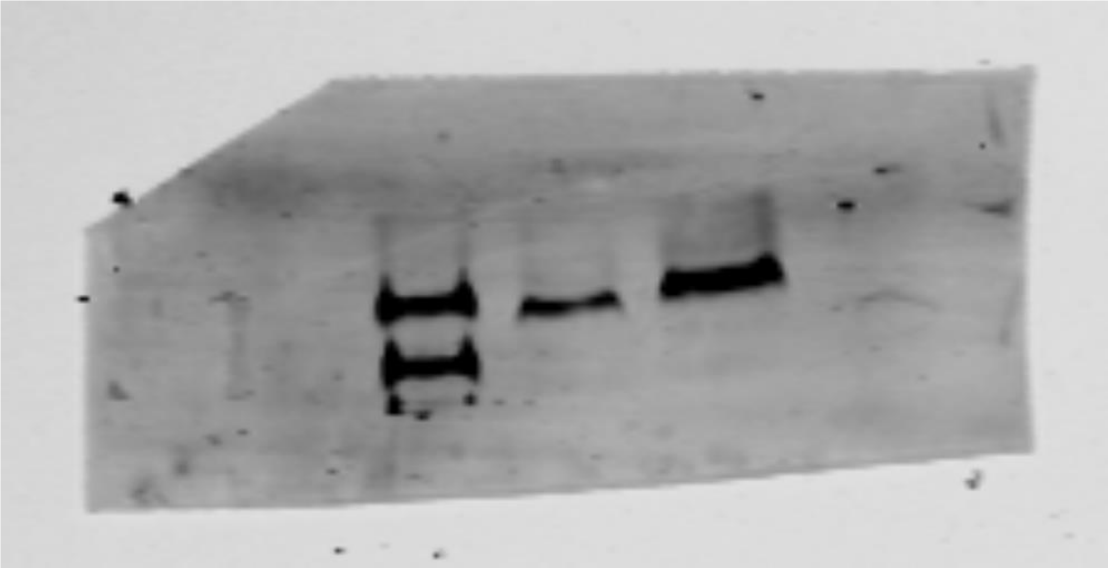

LNCaP AR

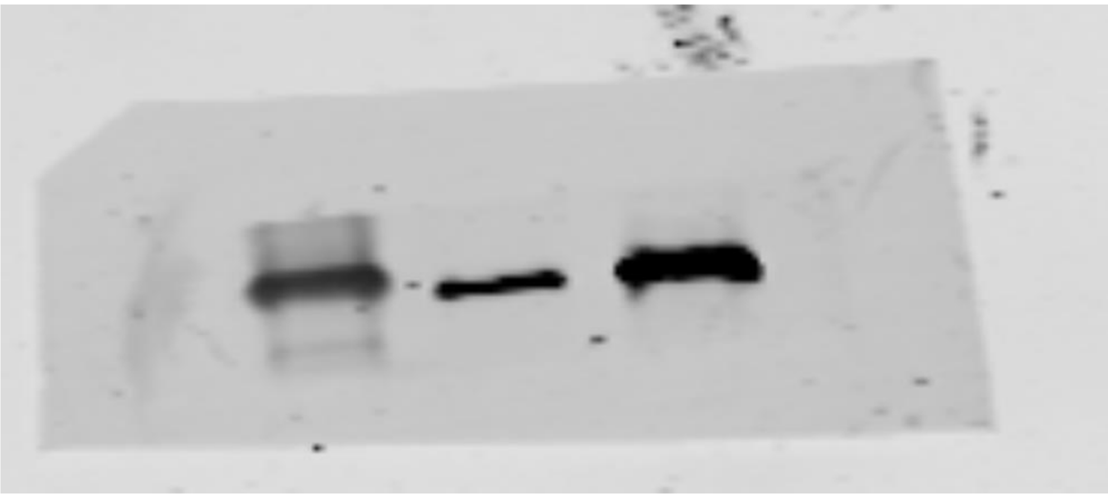

LNCaP PEX10

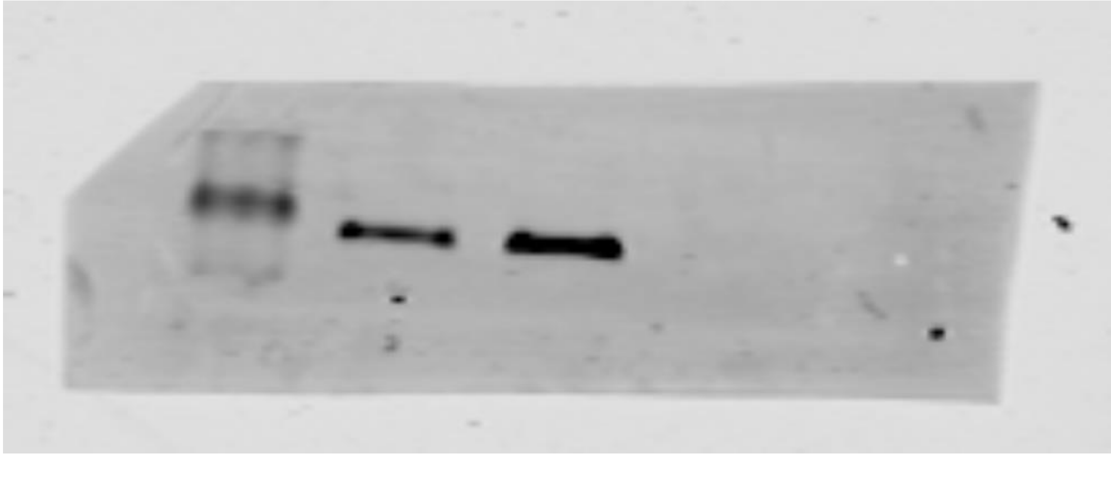

LNCaP MBOAT2

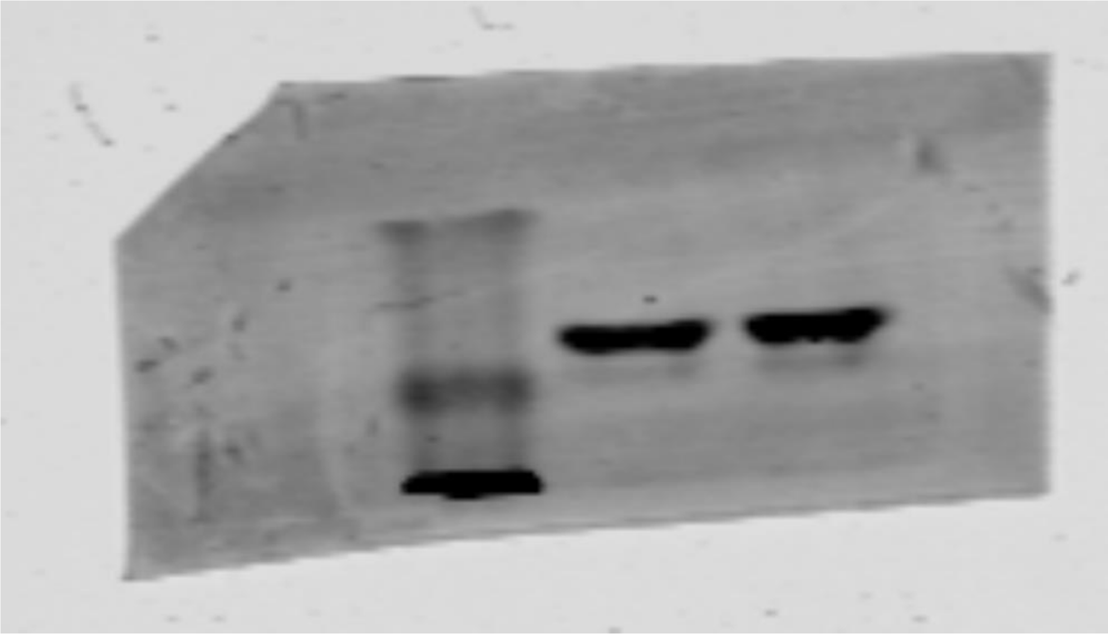

LNCaP GPX4

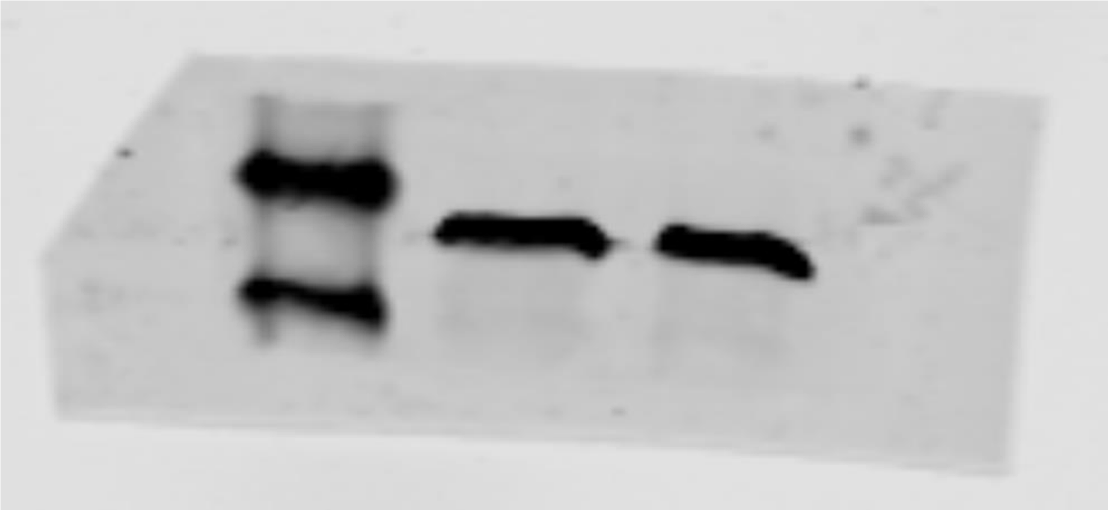

LNCaP β-Actin

Supplementary Figure 6E

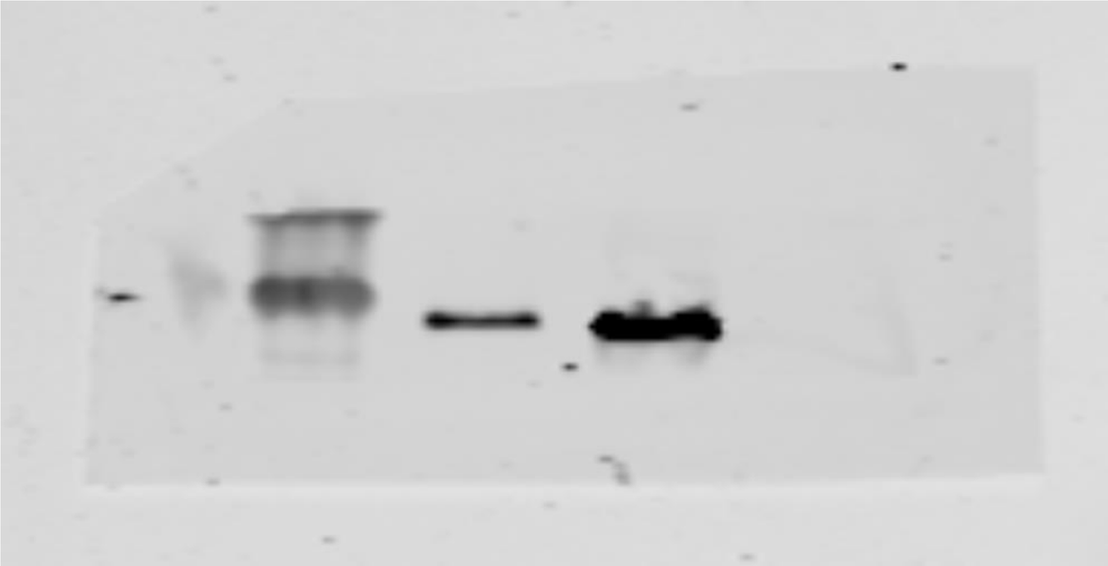

C4-2 PEX10

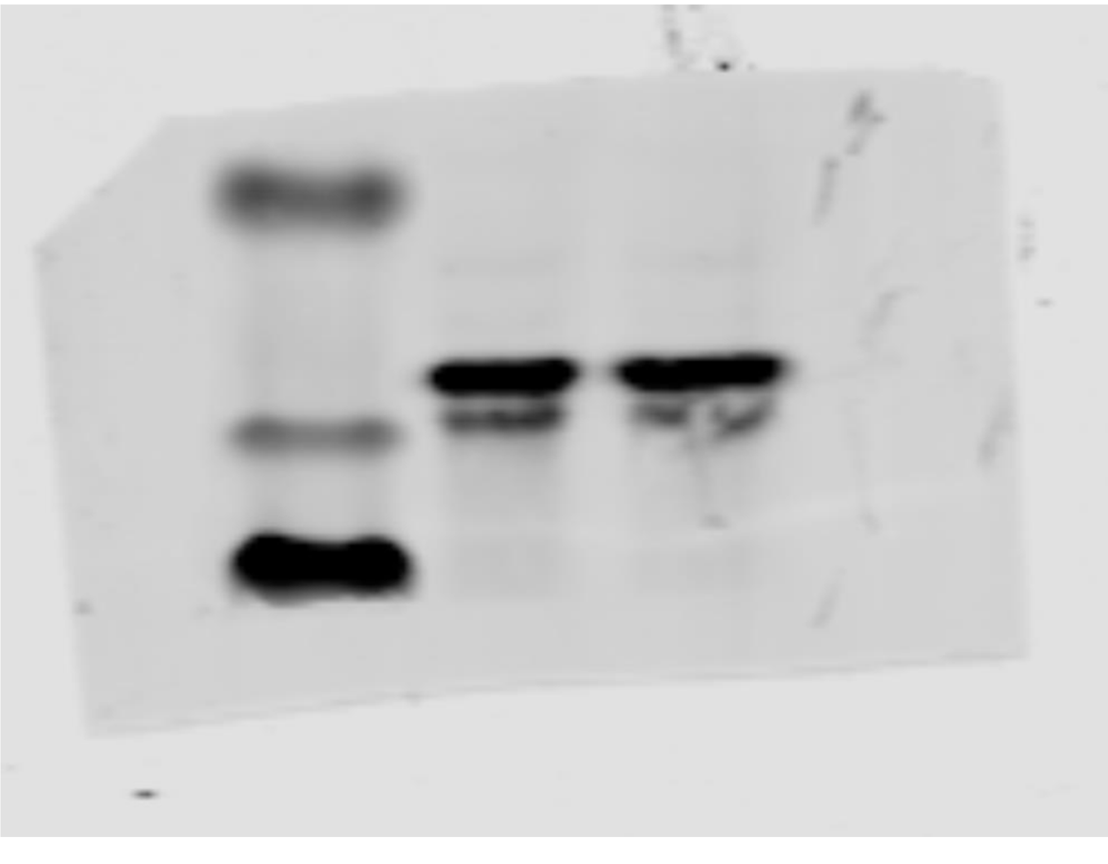

C4-2 GPX4

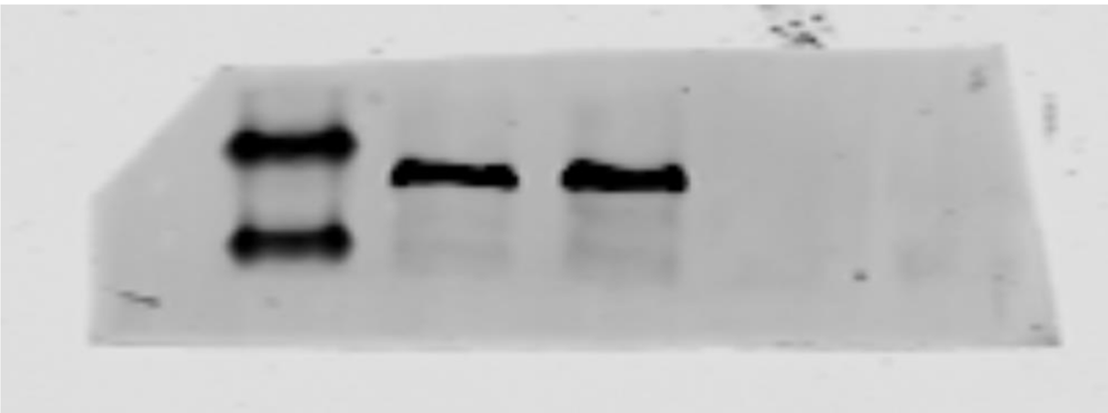

C4-2 β-Actin

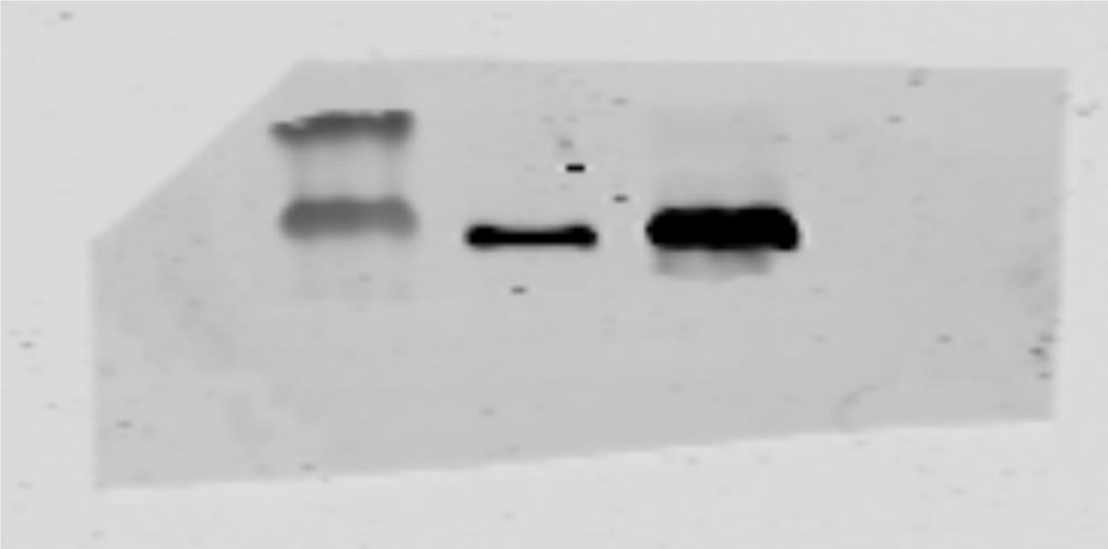

LNCaP PEX10

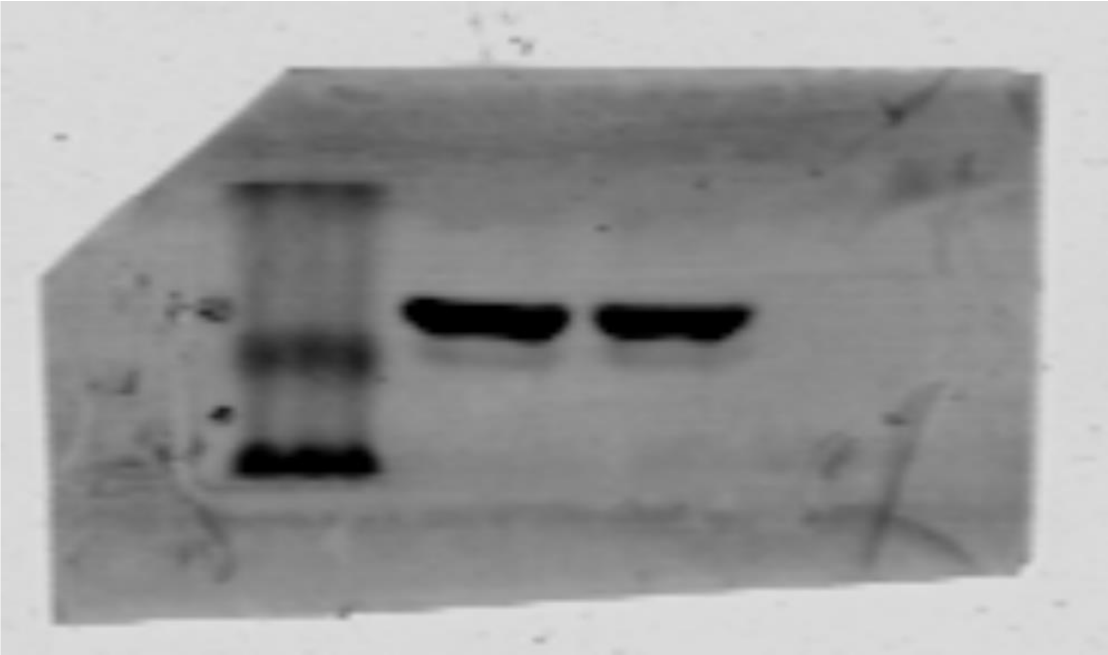

LNCaP GPX4

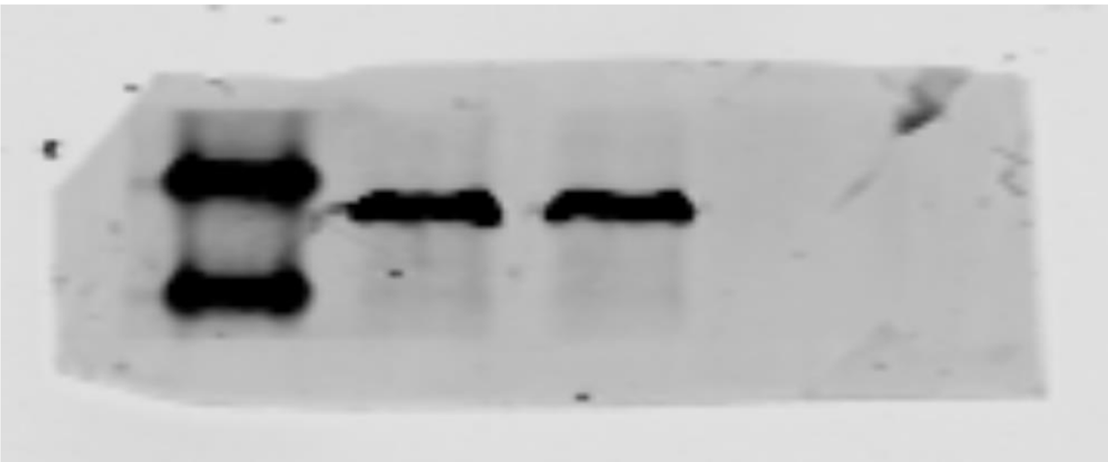

LNCaP β-Actin
